# Holling meets habitat selection: functional response of large herbivores revisited

**DOI:** 10.1186/s40462-021-00282-6

**Published:** 2021-09-06

**Authors:** Claudia Dupke, Anne Peters, Nicolas Morellet, Marco Heurich

**Affiliations:** 1 Department of Biometry and Environmental System Analysis, University of Freiburg, Freiburg, Germany; 2 Department of Visitor Managment and National Park Monitoring, Bavarian Forest National Park, Grafenau, Germany; 3 Chair of Wildlife Ecology and Management, University of Freiburg, Freiburg, Germany; 4 CEFS, INRAE, Université de Toulouse, Castanet-Tolosan, France

**Keywords:** Discrete choice, Herbivore, Landscape complementation, Landscape of fear, Mixed effects, Multinomial, Ungulate

## Abstract

**Background:**

Holling (Can Entomol 91(5):293–320, 1959) was the first to describe a functional response between a predator’s consumption-rate and the density of its prey. The same concept can be applied to the habitat selection of herbivores, specifically, the change in relative habitat use with the change in habitat availability. Functional responses in habitat selection at a home-range scale have been reported for several large herbivores. However, a link to Holling’s original functional response types has never been drawn, although it could replace the current phenomenological view with a more mechanistically based understanding of functional responses.

**Methods:**

In this study, discrete choice models were implemented as mixed-effects baseline-category logit models to analyze the variation in habitat selection of a large herbivore at seasonal and diurnal scales. Thus, changes in the use of land cover types with respect to their availability were investigated by monitoring 11 land cover types commonly used by roe deer (*Capreolus capreolus*) in the Bavarian Forest National Park, Germany. Functional response curves were then fitted using Holling’s formulas.

**Results:**

Strong evidence of non-linear functional responses was obtained for almost all of the examined land cover types. The shape of the functional response curves varied depending on the season, the time of day, and in some cases between sexes. These responses could be referenced to Holling’s types, with a predominance of type II.

**Conclusions:**

Our results indicate that Holling’s types can be applied to describe general patterns of the habitat selection behavior of herbivores. Functional responses in habitat selection may occur in situations requiring a trade-off in the selection of land cover types offering different resources, such as due to the temporally varying physiological needs of herbivores. Moreover, two associated parameters defining the curves (prey density and predation rate) can aid in the identification of temporal variations and in determinations of the strength of the cost-benefit ratio for a specific land cover type. Application of our novel approach, using Holling’s equations to describe functional responses in the habitat selection of herbivores, will allow the assignment of general land cover attraction values, independent of availability, thus facilitating the identification of suitable habitats.

## Introduction

Habitat selection studies have shed light on the mechanisms and driving forces underlying the spatial behaviors of animals [1]. Variations in the habitat selection of animals were linked to differences in the occupied environments, usually differences in resource availability [2]. Although landscapes are increasingly being characterized based on the climatic and environmental data collected using high-precision remote sensing [e.g. 3], many studies are still based on the assumption that habitat selection remains constant over the spectrum of available habitats (or resources) within an area [4, 5]. However, more than 20 years ago, Mysterud and Ims published a seminal paper showing that the use of a land cover type varies non-linearly with its availability, thereby merging the concept of functional response with the habitat use of animals within their home range third order selection, [6]. Subsequent empirical studies of different large herbivores within their home ranges confirmed the availability dependence of habitat selection [e.g. 7–11], such that the concept of functional response has been applied to both the habitat use and the habitat selection of herbivores [5].

Observations of functional response patterns within an animal population have shown that habitat selection behavior at the individual level changes in relation to the availability of land cover types within that animal’s home range, as the different land cover types are used for specific available resources, such as food or cover, related to an individual’s fitness [12, 13]. Land cover types differ in the structure of their vegetation and, from an animals’ perspective, in the cost-benefit ratio of their use, as use inevitably involves a trade-off between often opposing needs, such as protection against predators vs. food intake [14]. The strength of this trade-off depends on the resources offered by a given land cover type and affects the use of a land cover type with respect to its availability [15]. For example, the strength of a food/cover trade-off for moose (*Alces alces*) was linked to the strength of the functional response, such that functional response patterns were particularly pronounced during periods with the largest constraints, such as a high predation risk or the increase in energetic requirements necessitated by lactation [16]. Consequently, an animal’s functional response in habitat selection takes into account spatio-temporal variations in the perceived risk as well as the animal’s changing physiological requirements. Therefore, the functional responses of wildlife reflect the need to manage risk-resource trade-offs [14, 17], which will vary over time for the different land cover types.

Several methods to quantify variations in habitat selection with respect to environmental covariates are available [1, 18, 19], but the most widely employed is the analysis of resource selection functions [RSFs; 20], in which a combination of case-control-based sampling and logistic regression is used to relate the spatial use of animals to landscape characteristics [7, 21]. RSFs have been applied in different approaches aimed at identifying functional responses in habitat selection. For example, habitat selection coefficients can be estimated separately for individuals or groups with different habitat availabilities, with or without the inclusion of random effects [22, 23], although inclusion is strongly recommended [24]. Alternatively, habitat availability can be included as a fixed effect [11, 15, 25] or as an interaction with other covariates [26] to model the functional response explicitly. Functional response is usually presented as the effect of availability on either odds ratios [13, 15] or the selection coefficients [11, 23, 25]. For more formulations, see [5]. Although the relationships revealed by RSFs may indicate a functional response, they are difficult to interpret and quantify [4, 27, 28] because they represent selection for or against a certain habitat or resource unit but do not directly depict the relationship between availability and actual use [e.g., 15, 29].

Specifically, *use* cannot easily be inferred from a resource selection analysis because habitat selection is a function of and therefore dependent on availability [5]. Following Johnson (1980), *use* and *selection* are identical only in the theoretical case of equal availabilities of resource types. If the selection probabilities are averaged over all available units, probabilities of use can be obtained [29] and contrasted with the availability of a habitat or resource unit; however, no such a study is known to the authors.

The term functional response was introduced by Holling [30] who described the ability of carnivores, and specifically that of small insectivorous mammals, to handle and consume prey items according to the level of prey availability. Depending on the time required for searching, handling, and consuming individual prey, three types of functional responses curves depict the relationship between a carnivore’s ability to use prey items and prey availability: linear (type I), concave (type II), and sigmoid type III, [30]. Specialist carnivores were presumed to exhibit a type II functional response, i.e., the number of killed prey increases rapidly with prey density and levels off when search time and prey manipulation constrain the rate of prey consumption [31]. For a generalist predator, by contrast, a slower increase in the number of killed prey results in a sigmoid curve and thus a type III response, indicative of changes in the search target and foraging habitat as well as prey switching [32].

As defined by [30], functional responses take place at the finest scale of resource selection by an animal, i.e., at the within-habitat scale fourth order selection, [6]. Likewise, in herbivores, functional responses were initially studied at the bite level [33], with the amount of food ingested and processed by an animal related to food availability. The ability of herbivores to find, select, and process specific plants or their parts is similar to the ability of carnivores to search, hunt, and consume prey and is thus subject to behavioral constraints that generally manifest as type II functional responses [34].

Resource selection is a hierarchical process with respect to resource availability and the geographical range of a species. How food processing is scaled up from a fourth-order selection to higher spatial scales, such as the within-home range (third-order selection) and landscape scale second order selection [6], and the behavioral and physiological adjustments or movement constraints that may occur in the process, are insufficiently understood but see [27]. Here, we demonstrate that the equations underlying Holling’s types can also be used to model the functional response in habitat use as determined from field data for a theoretical approach, see [28], thus allowing a mechanistic rather than simply a phenomenological view of functional response.

Although Holling’s equations were originally developed for predator-prey systems, the occurrence of functional responses in other domains characterized by a saturation of consumption has already been found in other areas of biology, such as in the foraging of plants [35] and enzyme kinetics [36]. As an alternative method of assessing functional responses in habitat selection, we propose multinomial logit discrete-choice models [37], which take into account the multinomial structure of the data in analyses of use of specific choices. An advantage of this approach is the simple inference of functional response, because (proportional) use can be directly related to (proportional) availability, as originally proposed by Mysterud and Ims [38]. In this study, GPS locations of 36 free-ranging roe deer in the Bavarian Forest National Park, Germany were used to analyze the habitat selection of roe deer in 11 land cover types located within the home ranges of individuals. Specifically, we investigated how the use of a particular land cover type varied depending on its relative availability within the home range [38], taking into account temporal variations in use patterns at daily and monthly scales [15, 39] as well as differences between sexes [40–42].

The goal of this study was to relate the functional response curves of roe deer for each land cover type—both seasonally, i.e., during summer (June) and winter (December), and depending on the time of day, i.e., during day (noon) and night (midnight)—to Holling’s types. Based on previous studies of prey consumption by large mammalian carnivores [43, 44], we predicted that a non-linear relationship between the use of a land cover type and its availability (Holling’s type II or III) would prevail over use proportional to availability (Holling’s type I) (H1). Specifically, type II responses were expected more frequently in land cover types providing a specific resource, such as forage in meadows (H2) and type III responses in the use of difficult to access land cover types, e.g., disturbance areas, (H3). Our reasoning was that a land cover type in which movement barriers, such as deadwood, had to be overcome or required some form of adjustment would be used only if the availability of that type within the home range exceeded a certain threshold and therefore could no longer be avoided [25]. In addition, given the seasonal changes in the cost-benefit ratio of land cover types and their use, such as imposed by the changing physiological requirements of individuals, as well as the variations in the risk perceived by roe deer over time, such as human disturbances during daytime, the functional response was expected to differ both seasonally and over the course of the day, resulting in the need for roe deer to re-evaulate the risk-resource trade-off over time (H4) [14, 15, 45, 46]. Finally, we predicted that functional response curves would differ between sexes (H5) [41].

## Material and methods

### Study area and data

The study area encompassed the Bavarian Forest National Park (BFNP), located in south-eastern Germany along the Czech Republic-German border (center coordinates $49\,^{\circ }3^{\prime }19^{\prime \prime } N, 13\,^{\circ }12^{\prime } 9^{\prime \prime } E$). Along the park’s altitudinal gradient, three major forest types can be distinguished. Sub-alpine spruce forests of Norway spruce (*Picea abies*) and to a lesser extent mountain ash (*Sorbus aucuparia*) prevail above 1100 m (16% of the area). On slopes between 600 and 1100 m mixed montane forests containing Norway spruce, white fir (*Abies alba*), European beech (*Fagus sylvatica*), and sycamore maple (*Acer pseudoplatanus* predominate 68% of the area; [47]). In wet valley bottoms (16% of the area), often associated with cold-air pockets, Norway spruce, mountain ash, and birch (*Betula pendula, B. pubescens*) are the dominant trees [48]. Since the mid-1990s, massive bark beetle (*Ips typographus*) proliferations have occurred in the BFNP’s forests, resulting in a dieback of old spruce trees over an area of $\sim$7000 ha [49]. Multispectral aerial images of the study area from 2008 [50] were used to classify forest areas according to their land cover [51], which was grouped into 11 classes (see Table 1). Images with a resolution of 0.4 m were used to isolate vegetation against the background of other underlying surfaces, different kinds of vegetation, and different stages of vegetation of the same species [52].

The estimated roe deer population density in the BFNP is low, ranging between 1 and 3 animals/km$^2$ [53]. Hence, density was not expected to affect habitat selection behavior [25, 28]. From 2005 until 2012, a total of 168 roe deer were monitored. (details on roe deer capture and data processing are provided in Additional file 1: Appendix S1a). The final analysis consisted of 15,267 locations of 17 females and 19 males.

### Statistical methods

#### Process overview

For each individual, the relative availability of all land cover types in its home range was computed on a monthly basis (see Availability section). The model’s data set (see Modeling odds ratios) was generated by combining information on each position from the preprocessed GPS telemetry data with information on the used land cover type, the ID and sex of the recorded individual, the time of day (hour), and the month of data collection. The data set was then divided into ten subsets, one for each land cover type, except the reference type. The subset for landscape type *i* contained only those entries when it was used plus the entries when the reference type was used. For each subset, the response variable *present* was added, which was 1 if the respective land cover type was used and 0 if the reference type was used. A column for the value of relative availability, unique for a specific individual and month, was included. In cases in which $present=0$, the value assigned to availability corresponded to the availability of the focal land cover type (not the reference type) in the monthly home range of the recorded individual.

The ten data sets were used to build ten independent logistic regression models that included hour, month, relative availability, and the sex and ID of the individual as explanatory variables. Predictions of these models (on the link scale) provided the log odds ratios (ORs), an indicator of the likelihood that animals select land cover type *i* over the reference type if they are equally available. The values of the ORs were used to derive the choice probability, by applying the multinomial logit link (see Multicategorical logit model). The choice probability $\pi _i$ is the probability that the used land cover type is type *i*. Since the logistic regression models included the time of day and the time of year (month), the ORs were calculated separately for each time slot (hour, month) and therefore so were the choice probabilities.

In our study, the choice probability was equivalent to the proportional use of a land cover type (see Terminology). Proportional use was predicted over the observed ranges of availability for all land cover types for a specific time slot. These curves were used to determine the optimal Holling’s function.

#### Availability

Availability was assessed for each individual per month and was calculated as a 95% minimum convex polygon around an individual’s locations on a monthly basis using the R-package *adehabitatHR* [54]. Data from individuals with a biologically unrealistic home range size, due, for example, to dispersal behavior, were discarded, using the 90th percentile as a cut-off value (for females: 182 ha, for males: 459 ha). A rasterized landscape with a grid cell size of $10\times 10 \mathrm{m}^2$ was used to obtain the proportion of each land cover type in the collection of available land cover types within the monthly home ranges referred to as the *relative availability* or the *proportion available* [55].

#### Modeling odds ratios

Ten logistic regression models were fitted that estimated the OR of using a land cover type versus the reference type. The land cover type with the highest prevalence was chosen as the baseline category [sensu 56], which in the BFNP was the old mixed stand, used in 27% of all recorded locations. The data set for the *i*th model was limited to observations from land cover type *i* and the baseline category *K*, where the binary response variable was $Y=1$ if an individual was observed in land cover type *i* and $Y=0$ if it was observed in the reference land cover type *K*. Hence, no pseudo-absence points needed to be generated.

The models $f_i(\varvec{x} = \alpha _i + \varvec{\beta _i} \varvec{X_j} + f_{i1}(hour, month) + f_{i2}(rel.availibilty) + log(rel.availability) + \epsilon _{ij} + \gamma _{iyear}$, $i = 1, \ldots , K-1$ included the possible effects of time (*hour*, *month*), sex, season, and the relative availability of the land cover type. The relative availability of a land cover type *i* varied across the home ranges of different animals *j* and depended on the time of year (*m*, month). This variation was accounted for by including the logarithm of the relative availability of a land cover type within the corresponding monthly home range of the animal *j* as an offset term “base rate”, [1].

The offset reflected the assumption of a linear increase of the OR with increasing availability. Any deviation from this direct proportionality with factor 1, and hence, any form of a functional response [38], was detected by including either a parametric or non-parametric (smoothing spline) effect of availability. Whether the type of functional response varied depending on the time of year (on a monthly basis) and with respect to the individual’s sex was then investigated. The smoothness of the spline functions over the range of availability was controlled for by setting the smoothing parameter to $\lambda =2$ [57, p.128].

Temporal variation in selection behavior was accounted for by including a term for time $f_{i1}(hour, month)$, modeled by a cyclic (tensor product) smooth function, with or without distinguishing between the sexes [58]. Although, for the sake of simplicity, neither further covariates, such as continuous variables (e.g. distance to roads), nor random slopes were included in the model formula, extensions are technically feasible [56]. Variations in selection behavior across individuals and years were considered by including simple random effects $\epsilon _{ij}$, $\gamma _{iyear}$ on the intercept for individuals and years, to enable the prevalence to vary between individuals and year [8].

Because random slopes are technically integrable and allow coefficients to vary randomly across individuals according to some continuous distribution, their inclusion in the models, if possible, is highly recommended [24]. However, in our models there was no variable for which a random slope would have been reasonable.

#### Model selection and model fit

Eighteen different models $f_i(\varvec{x})$ estimating the effects of the above mentioned variables on the ORs were estimated for each land cover type $i = 1, \ldots , K$, whereby the focus was on the varying effect of relative availability. To take into account the problem of over-fitting, the prediction performance of all models was measured by applying a ten-fold cross-validation (for details, see Additional file 1:Appendix 1b).

#### Multicategorical logit model

As our aim was to analyze the choice behavior of animals confronted with a discrete set of options, i.e., 11 land cover types, the multinomial structure of the data was taken into account by the application of discrete-choice models [59, 60], since the probabilities of choosing one of the land cover types at a specific time $P(Y=i|\varvec{x_t}) = \pi _i(\varvec{x_t})$ must sum to 1, $\pi _{1}(\varvec{x}_t)+\cdots +\pi _{K}(\varvec{x}_t) = 1$ for any $\varvec{x_t}$. The fitted logistic regression models explained above describe how strongly the use of a specific land cover type deviates from the use of the reference type (over the range of observed availability and over time). As all models referred to the same reference or baseline category, we refer to them in the following as baseline-category logit models [56]. The next step was to calculate the use distribution [55] by using the multinomial link.

The baseline-category logit models model the variation of the odds; in this case, the probability of selecting land cover type *i* divided by the probability of selecting the reference type *K* for an animal encountering land cover type *i* or *K*. Hence, predictions of these baseline-category logit models for specific $\varvec{x_t}$ on the link scale provide the log OR $log \frac{\pi _i(\varvec{x_t})}{\pi _K(\varvec{x_t})} = f_i(\varvec{x_t})$. Based on the log OR $f_i(\varvec{x_t})$ for all land cover types, derived as explained above, the *use distribution* was estimated using the multinomial logit link [56]:

1 $$\begin{aligned} \pi _{i}(\varvec{x_{tj}})= \frac{exp(f_i(\varvec{x_{tj}}))}{1+\sum _{s=1}^{K-1} exp(f_s(\varvec{x_{tj}}))} = \frac{\alpha _i + \varvec{\beta _i} \varvec{X_{tj}} + \cdots + \epsilon _{ij} + \gamma _{iyear}}{1+\sum _{s=1}^{K-1} (\alpha _s + \varvec{\beta _s} \varvec{X_{tj}} + \cdots + \epsilon _{sj} + \gamma _{syear})} \end{aligned}$$

for each land cover type $i = 1, \ldots , K$, for animals *j* at time *t*. $f_K$ is 0 for identifiability reasons. The denominator of Eq. 1 was the same for all land cover types *i* at a specific time *t*. For the predictions, it was ensured that the sum of the values for relative availability over all land cover types equaled 1.

#### Terminology

The discrete choice model provides the probability that a certain land cover type is chosen over the other available land cover types. This so-called choice probability depends on the probability of selection and on the proportion of the other available types in the choice set. The model implicitly assumes that all units in the choice set (here, land cover types) are equally reachable by the individuals. Since the time interval between two recordings was > 25 h, it was assumed that every location in the home range could be reached. As the entire study area was categorized into discrete land cover types, the choice set was identical to the set of available units and therefore the choice probability was identical to the use distribution [55]. In our study, because the choice set consisted of categorical land cover types, the use distribution was equivalent to proportional use [55].

In the following, the term *selection* refers to the action that a resource unit (or land cover type) is selected independent of its availability in the area when it is encountered by an animal [55].

#### Referencing Holling’s types in the context of habitat selection

Predictions of the proportional use of all land cover types were made over the observed range of relative availability. The functional response curves of roe deer were linked to Holling’s types by estimating an optimal fit for each curve relating proportional use to the availability of a land cover type based on Holling’s equations [30]. Theoretically, this could have been done for all times of year and day for each land cover type. However, we focused on specific times, namely, summer (June) versus winter (December), and on day (noon) versus night (midnight). For type I: $h_I(x)=a x$, where *x* is the relative availability of a land cover type (a value between 0 and 1), and *a* the proportionality factor; then for type II: $h_{II} (x)=\frac{ax}{b+x}$; for type III: $h_{III} (x)=\frac{ax^2}{b^2+x^2}$. The optimization routine minimized the residual sum of squares between the values of $h_I(x)$, $h_{II}(x)$ and $h_{III}(x)$ and the values of $\pi _{i}(\varvec{x})$ (Eq. 1) estimated from the multicategory logit models by finding optimal values for *a* and *b*. The R function *optim* of the package *stats* [61] was used for this purpose, with the values of *a* and *b* limited to 0 and 1 for both $h_{II}$ and $h_{III}$. The Holling type with the smallest residual sum of squares was considered to best explain the shape of the obtained functional response curve.

The most familiar Holling’s type is the type II functional response that is mathematically equivalent to the Michaelis–Menten equation [36]. The Holling type describes predator-prey-dynamics, and the Michaelis and Menten equation enzyme kinetics using the same formula [35]:

2 $$\begin{aligned} y(x)=\frac{ax}{b+x} \end{aligned}$$

where *x* is the number of prey (or amount of substrate) available to a predator (or enzyme), *y*(*x*) is the predation rate (or reaction rate), and the parameters *a* and *b* describe the functional relation between prey density (or substrate concentration) and predation rate (or reaction rate). In the equation, *a* is the maximum value (upper limit) of the number of prey (or substrate) a predator (or enzyme) can handle. In fact, *a* is an asymptote, which means that as *x* becomes larger its value is approached but is never reached. The value *b* is the Michaelis–Menten-constant ($K_m$), i.e., the substrate concentration at which the enzyme reaction rate is half of the maximum rate. In chemistry, *a* and *b* are used to characterize an enzyme-substrate-complex; in particular, $K_m$ is a measure of the affinity of an enzyme for a particular substrate.

**Fig. 1:**
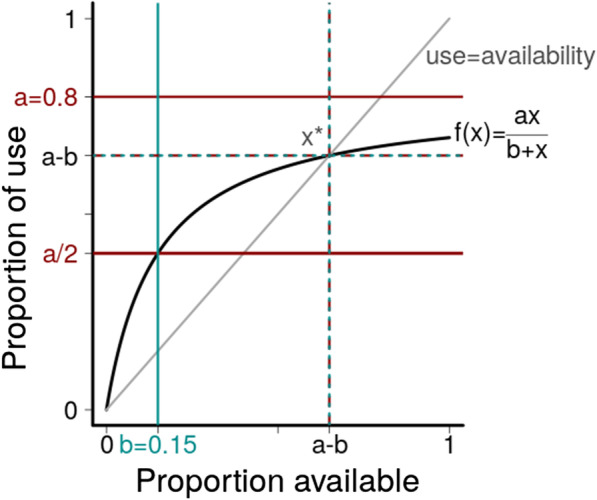
Concept plot of the most familiar Holling’s type II functional response $y(x)=\frac{ax}{b+x}$ for $a=0.8$ and $b=0.15$. *x* is the proportion of availability of a habitat in the home range, limited between 0 and 1, *f*(*x*) is the use of a habitat limited between 0 and *a*, the upper bound of use and *b* the availability of a habitat at which the habitat is used half of the maximum ($f(b)=\frac{a}{2}$). Parameters *a* and *b* become ecologically valueable and interpretable when applying limit calculations (see Additional file 1: Appendix S2, S6)

Given the Michaelis–Menten equation (2) in the context of habitat selection behavior, *x* is the proportion of availability of a habitat in the home range, limited to between 0 and 1, *y*(*x*) is the proportional use of a habitat, also limited to between 0 and 1, *a* is the maximum proportional use, and *b* is the availability of a habitat at which the habitat is used half of the maximum use ($y(b)=\frac{a}{2}$, Fig. 1). While the latter parameter may be rather elusive, *b* and *a* become ecologically valuable and interpretable if limit calculations are applied:

- if $b \rightarrow 0$, then proportional use $\rightarrow a$
- if $b \rightarrow 1$, then proportional use $\rightarrow \frac{a}{2}$
- if $a \rightarrow 0$, then proportional use $\rightarrow 0$

For small *x* compared to *b*, *y*(*x*) converges to *aN*/*b*, based on a linear slope for a small value of *x*. However, for a larger *x*, *y*(*x*) converges to *a* and *b* is neglibible. Furthermore, for given parameters *a* and *b*, the value of availability *x* when availability equals proportional use can be derived from the Eq. (2). If $y=x$ is inserted in the Eq. (2), then proportional use equals availability if $x=a-b$ (and of course, if $x=0$, Additional file 1: Appendix S2). It is clear that as *b* becomes smaller so do the availabilities at which proportional use reaches its saturation. Thus, the smaller the value of *b* (minimum 0), the faster the increase in proportional use with increasing availability. The larger the value of *b* (maximum 1), the slower the increase in proportional use with increasing availability. Furthermore, if $a>b$, $x=a-b$ is the availability of a habitat at which a switch in use occurs, from being disproportionately high to being disproportionately low. Consequently, for $b>a$, there is no disproportionally higher use at all, but instead a disproportionally lower use for all availabilities in the range of 0–1.

A similar relation between *a* and *b* applies in functional type III. The value of availability when availability equals proportional use is $x_{1,2}= \frac{a}{2} \pm \sqrt{\left( \frac{a}{2}\right) ^2-b^2}$ (for the derivation, see the Additional file 1: Appendix S2). This implies that *b* must be $< a/2$ to ensure that there is such a point. However, $b>a/2$ implies it can be derived that a habitat is used disproportionally less than its availability over the entire range of availability. In addition, the inflection point $b/\sqrt{3}$, defined as the value of availability when the curve changes from convex to concave, can also be calculated (see Additional file 1: Appendix S2 for a derivation). Ecologically speaking, for values greater than this proportion of availability, selection is higher than in the case of availabilities below the inflection point and thus, relative use is disproportionately higher than relative availability.

Consequently, the parameters of Holling’s equation can be used to identify the attractivity of a habitat for animals (Table 2).

The methods described herein are implemented and available in the R-package FunResp with enclosed vignette [62, Additional file 3]. A simulation study in Additional file 2: Appendix S3 provides a methodological overview of how Holling’s types and habitat selection analysis can be linked to analyses of habitat selection.

## Results

**Fig. 2:**
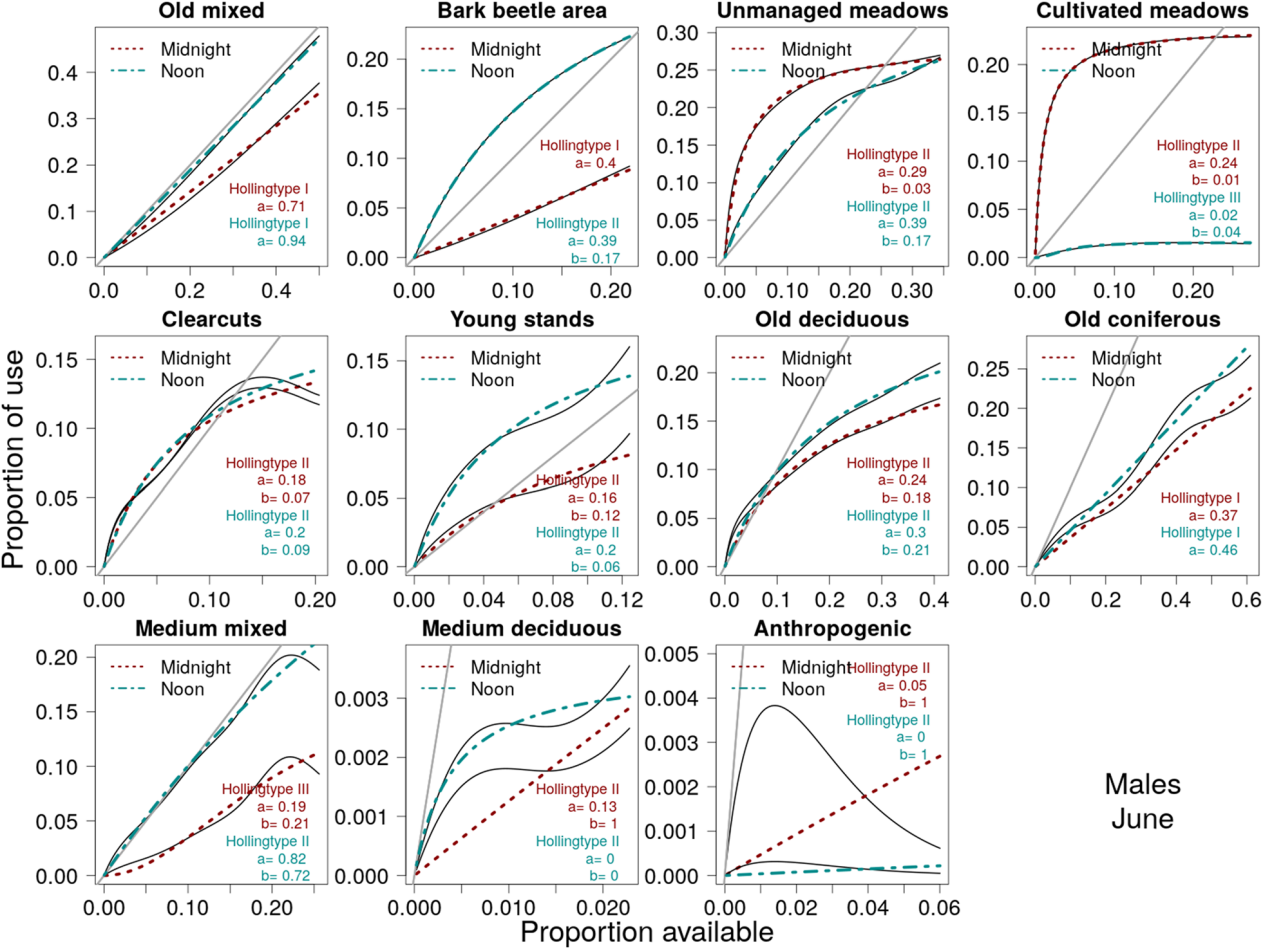
Shapes of functional response curves based on Holling’s types I, II or III for all habitats in June for 19 males roe deer during night (red dashed line) and day (green dot-dashed line) and the associated estimated optimal values for the parameters defining the Holling type. Black lines in the background of the coloured curves are the estimated proportional use based on multicategory logit models. Grey line indicates proportionality of use to availabilty (absence of functional response)

A general behavioral pattern observed in roe deer in the BFNP was a change in their habitat use based on the availability of a land cover type (Fig. 2). Functional responses to almost all land cover types were clearly determined, as the estimated proportional use curves differed strongly from straight lines (Fig. 2). The exception was the use of old mixed stands, especially during winter, in which case use equaled availability (Fig. 2). Basically, three main patterns emerged (Fig. 2): an approximately linear relationship (Holling’s type I), a concave curve (type II), and a sigmoid curve (type III).

A type II response indicates increasing use with increasing availability, but a decrease in the selection probability with greater availability (whereby selection is the use of a land cover type if it is encountered). The proportional use of a land cover type varied distinctly between seasons and depending on the time of day, as did the parameters *a* and *b*. Type II responses were particularly pronounced for land cover types primarily offering a single resource (Table 1), such as food in the case of meadows.

The estimated maximum proportional use *a* was typically much greater than *b* (Additional file 1: S4, S5). If *a* is larger than *b*, the use of a specific land cover type is disproportionately high as long the availability is less than $x^*= a-b$ ( Fig. 1, for a derivation, see Additional file 1: S2). The value of *b* indicates the strength of the proportional use irrespective of availability, with a smaller *b* corresponding to a greater proportional use for small values of availabilities. Hence, the value of *b* provides information about how fast the functional curve reaches saturation and is an indicator of the attractiveness of a land cover type. The value of *a* quantifies the potential maximum proportional use of a land cover type but it must be regarded in light of the value of *b* (for details, see Additional file 1: S6).

For instance, the probability of males using cultivated meadows in summer increased strongly with increasing availability for very low values of availability (< 0.03), with a very steep slope; however, for higher values of availability (> 0.1), the slope was almost 0 (Fig. 2). This high use for small values of availability was reflected in the small value of *b* (Table 3). For cultivated meadows in summer at midnight, $b=0.01$, resulting in a very steep increase in the functional response curve. Compared to unmanaged meadows, in which $b=0.03$, this increase was less pronounced at midnight and especially less pronounced at noon for $b=0.17$. For many of the land cover types (e.g., clearcuts, young stands, old deciduous, and medium mixed stands, Table 3), there were large variations in the *b*-value for different times of day and year but also between males and females.

If $b<a$, the value of *b* influences when the shift occurs from selective use to avoidance, which is the tipping point $x^* = a-b$. This value differed greatly between different land cover types but also within land cover types for different times of day and year (Table 3). For males in summer at midnight unmanaged meadows were frequently used (proportional use > proportional availability) when availability was less than $x^* = 0.26$ and were avoided (proportional use < proportional availability) when the availability of unmanaged meadows exceeded $26\%$ of the home range’s area. In summer at noon, the tipping point was smaller ($x^* = 0.22$), indicating that the use of unmanaged meadows was less at noon than at midnight (Table 3).

## Discussion

As predicted by our hypotheses, the proportional use of a land cover type by roe deer was well described as functional responses, with distinct variability in the shapes of those responses among land cover types and depending on the month, time of day and, in a few cases, an individual’s sex. Thus, our results were consistent with a non-linear relationship between proportional use and the availability of a land cover type, as this pattern occurred in most of the considered land cover types. Furthermore, the shapes of the curves could be linked to Holling’s equations and the functional response parameters in turn estimated. By comparing those parameters with respect to the time of day, the season, sex, and different land cover types, our approach sheds light on the temporal variations in the use of land cover types by large herbivores.

The parameters describing the functional response curves can be interpreted ecologically using the same terms applied by Holling to prey-predator-systems [63]. When transferred to the habitat use behavior of animals, these parameters provide information about the functional relationship between habitat availability and proportional use, and especially about general habitat preference or avoidance behaviors either as a function or independent of habitat availability. For a type I functional response, the slope describing the increase in the predation rate with increasing prey density (*a* in $h_I(x)=a x$) is also relevant to habitat use, as it represents the linear increase in the proportional use of a resource unit with the latter’s increasing availability.

For type II and type III functional responses, $h_{II} (x)=\frac{ax}{b+x}$ and $h_{III} (x)=\frac{ax^2}{b^2+x^2}$, *a* is the asymptote that is approached as *x* increases. While it originally represented the maximum number of prey a predator can handle per unit time, when applied to habitat use it indicates the theoretical maximum use of a resource unit at a specific time (season and time of day). The value of *b* was originally defined as the prey density at which a predator exhibits half of its maximum predation rate, which is associated with the handling time. In the case of habitat use, value of *b* is the availability at which a resource unit is used at half of its theoretical maximum use during a specific time. Thus, the smaller the value of *b* the greater the attraction of the resource unit for an animal, as the functional response curve becomes saturated for very small values of availabilities. Consequently, in the context of habitat use, *b* is associated with the attractiveness of a resource unit for a species.

A type II functional response, indicative of a continuous decrease in the probability of selection with increased availability of a resource unit, was the most commonly observed pattern, especially in summer (Fig. 2). However, the upper limit of proportional use (denoted by *a*) and the degree of attraction (denoted by *b*) differed between different land cover types and over different time scales.

Habitat use by herbivores can involve a trade-off between conflicting needs, such as food intake and risk avoidance, which affects the proportion of time the animals spend using a resource unit [15, 58]. For instance, the high availability of forage biomass offered by meadows can satisfy the energy demands of roe deer but the lack of cover implies a higher risk [64]. By contrast, habitats such as medium-aged mixed stands offer shelter but their forage biomass is of lower quality and quantity [Table 3; 10, 65]. Consequently, the trade-off between conflicting needs will be perceived differently by an animal depending on the land cover type, such that stronger perceived trade-offs will result in more pronounced functional response curves [16]. Hence, risk-resource trade-offs may manifest as a functional response in habitat use [15, 38] and insights into the nature of those trade-offs can be gained by determining the Holling’s type and associated parameters *a* and *b*.

For the studied roe deer in the BFNP, type II functional response patterns indicated the saturation of proportional use for relatively small values of relative availability for some land cover types. This was demonstrated by the preference of males for cultivated meadows (Fig. 2) in summer during the night, as the use of this land cover type strongly increased for very low availabilities (half maximum use at availability $b = 0.01$) but remained almost constant for as availability increased, with an upper limit of $a = 0.24$. Thus, male roe deer did not spend more than one fourth of their time in a meadow during the night, but it was apparently enough to satisfy their needs; this pattern did not change when the availability of cultivated meadows within the home range increased further, consistent with the strong trade-off that confronts animals using this land cover type [16]. The ratio *a*/*b* (Table 3, Additional file 1: Appendix S6) describes the strength of the functional response: the larger the ratio, the greater the overall proportional use. A ratio > 1 implies that proportional use exceeds proportional availability, resulting in a higher level of selection probability for availabilities less than $x^* =a-b$. A small *a*/*b* ratio indicates a less pronounced or even an absent functional response as found for land cover types with a low food vs. cover trade-off, such as medium-aged mixed stands (Table 3, Fig. 2).

In type III responses, proportional use is low at low availabilities but then increases rapidly. The presence of land cover types evoking a type III functional response suggested that in those cases availability influenced habitat use to a larger degree than did an individual’s physiological needs. In predator-prey dynamics, a type III response occurs in systems with more than one prey species and can be explained by either a learning process or prey switching [34, 66]. In the context of habitat use in herbivores, a type III response may occur if an individual has no other option than to become accustomed to a habitat type.

In our study, in land cover types that provided a lower diversity of resources, such as cultivated meadows, with their large amounts of forage but little cover, strong satiation occurred even at low levels of availability. In meadows, the deer’s focus on forage rather than on cover resulted in a functional response curve resembling Holling’s type II [30], indicating that once an animal has fulfilled its need for forage, it switches to another land cover type, one that offers a different resource than meadows. This finding agrees with a previous study of the foraging strategy of ungulates: having filled their stomachs, the animals moved to another, safer place to chew and ruminate [67]. However, for land cover types that provide both food and cover, such as young stands, their use by roe deer was only slightly attenuated with increasing availability, as the deer were not focused on a single resource [25]. These findings imply the presence of landscape complementation sensu [68] involving different land cover types, which can impact habitat use because roe deer must vary their use of different land cover types to obtain the resources needed to meet their current physiological needs [41, 69].

In land cover types mainly used for foraging, the time spent therein should have a strongly positive correlation with forage intake. Accordingly, due to the strong correlation in grazers between bite size and biomass intake, saturation should be more pronounced in land cover types mainly used for foraging but otherwise containing less diverse resources. For roe deer, as a browser, bite size depends on leaf size [70]; thus, the relationship between the time spent in a land cover type and the amount of biomass intake will not necessarily be linear. Instead, the time needed for a deer to fill its stomach will depend on the detection and accessibility of suitable forage, which can strongly vary between different land cover types. When suitable forage is abundant and the detection time is therefore low, as in meadows and clearcuts, use would be directly related to forage intake, as shown for grazers.

This study was based on a data set of roe deer from a specific study region, namely, the BFNP. While habitat selection is a complex process, involving a wide range of variables, our approach can be applied to detect general trends in habitat selection, thus fostering the development of effective large-scale wildlife management and protection measures. However, we emphasize that the two-step process described herein has the disadvantage that the uncertainty, in the form of confidence intervals, is difficult to calculate. To obtain functional response curves that include confidence intervals, the multinomial regression could also be estimated using the R package mgcv [57], whose confidence intervals tend to be smaller than those obtained with baseline category logit models [56]. Subsequently, the optimal Holling type and its associated parameters could be estimated using maximum likelihood methods instead of the least-squares method, such that uncertainties in the estimation could be quantified. In principle, bootstrap methods can also be used to account for imprecision. Even more welcome would be Bayesian models, for which a full hierarchical model would be created, that already contain Holling’s functional curves. This would overcome the two-step process and quantify the uncertainty in parameter estimation.

**Table 1:** Overview of the relative availability of habitat types in the study area, the relative availability within the home ranges, and the relative use of those habitat types by roe deer in the Bavarian Forest National Park, in descending order of use

Habitat	Availability in the study area	Availability in the home range	Proportional use	Cover	Biomass ($\mathrm{gm}^{-3}$)
Old deciduous	0.184	0.230	0.247	0.77 (0.31)	45 (79)
Old mixed	0.231	0.393	0.188	0.73 (0.34)	54 (75)
Old coniferous	0.220	0.188	0.170	0.63 (0.35)	24 (58)
Cultivated meadows	0.051	0.060	0.102	0.16 (0.2)	299 (91)
Medium mixed	0.075	0.045	0.079	0.67 (0.33)	85 (80)
Unmanaged meadows	0.020	0.014	0.064	0.31 (0.26)	349 (41)
Clearcuts	0.046	0.024	0.062	0.25 (0.24)	29 (57)
Young stands	0.020	0.009	0.047	0.36 (0.27)	54(-)
Anthropogenic	0.027	0.022	0.018	0.36 (0.33)	300 (-)
Medium deciduous	0.015	0.007	0.014	0.66 (0.36)	89 (114)
Disturbance area	0.111	0.008	0.010	0.31 (0.24)	10 (53)

The values for cover are the means (and standard deviations) of the fractional cover above 2 m over the study area, as derived from high-resolution airborne laser-scanning (LiDAR, light detection and ranging) in summer. Biomass is the average value of dried biomass within 1 $m^3$ of a habitat type adapted from [58]

**Table 2:** Interpretation of the parameters in Hollings’ equations of functional response types I, II and III

Holling type	Use<availability for the entire range of availability if	Use $=$ availability for
I	$a<1$	−
II	$b>a$	$x=a-b$
III	$b> a/2$	$x_{1,2}= \frac{a}{2} \pm \sqrt{\left( \frac{a}{2}\right) ^2-b^2}$

*a* is the maximum proportional use of a habitat, and *b* the availability of a habitat at which the habitat is selected with half of the maximum probability. These conditions allow a determination of whether the proportional use of a resource unit is disproportionally low (proportional use < proportional availability) over the entire range of availability. If this condition does not hold, the value of availability at which it equals the proportional use can be determined

**Table 3:** Parameters estimated for Holling’s equations fitted to the functional response curves describing the use of the available habitat types by male roe deer in the Bavarian Forest National Park in summer (June) at different times of day (noon/midnight)

Habitat	Sex	Month	Hour	type	a	b	*a*/*b*	$x^{*}$
Old mixed	m	6	0	I	0.71			0
			12	I	0.94			0
Bark beetle area	m	6	0	I	0.40			0
			12	II	0.39	0.17	2.34	0.23
Unmanaged meadows	m	6	0	II	0.29	0.03	9.16	0.26
			12	II	0.39	0.17	2.31	0.22
Cultivated meadows	m	6	0	II	0.24	0.01	22.67	0.23
			12	III	0.02	0.04	0.40	0
Clearcuts	m	6	0	II	0.18	0.07	2.51	0.11
			12	II	0.20	0.09	2.35	0.12
Young stands	m	6	0	II	0.16	0.12	1.35	0.04
			12	II	0.20	0.06	3.52	0.15
Old deciduous	m	6	0	II	0.24	0.18	1.32	0.06
			12	II	0.30	0.21	1.45	0.09
Old coniferous	m	6	0	I	0.37			0
			12	I	0.46			0
Medium mixed	m	6	0	III	0.19	0.21	0.90	0
			12	II	0.82	0.72	1.14	0.10
Medium deciduous	m	6	0	II	0.13	1.00	0.13	0
			12	II	0.00	0.00	0.87	0
Anthropogenic	m	6	0	II	0.05	1.00	0.05	0
			12	II	0.00	1.00	0.00	0

Associated curves are shown in Fig. 2. Holling’s equations for type I $h_I(x)=a x$, where x is the availability of a habitat; for type II: $h_{II} (x)=\frac{ax}{b+x}$ and for type III: $h_{III} (x)=\frac{ax^2}{b^2+x^2}$. The fraction $\frac{a}{b}$ indicates the selection strength independent of availability of a habitat: the greater the value the greater the general use. The value $x^* = a-b$ for Holling type II is the availability when use equals availability, hence the value of relative availability at which no selection occurs, which is the tipping point at which habitat selection switches to habitat avoidance (Fig. 1)

## Conclusions

From an ecological perspective, the parameters *a* and *b*, for estimating maximum proportional use and the attractiveness of a habitat, respectively, can shed light on general trends in habitat selection or avoidance. The approach introduced herein, using multicategorical logit models and relating the derived functional response curves to Holling’s types, can facilitate the detection of general trends in habitat selection and avoidance, by replacing a phenomenological view of functional responses with a more mechanistic view. Application of the equations underlying Holling’s functional response types to the habitat selection behavior of animals enables estimates of the relevance of a particular habitat type for the species of interest, thus shedding light on the cost-benefit ratio as perceived by animals using this habitat. A further advantage of this approach is that it can improve comparisons of the habitat selection behavior of animals in different study areas).

## Supplementary Information

**Addtional file 1**: Appendix S1a: Roe Deer Capture and Radiotelemetry Data. Appendix S1b: Model selection and model fit. Appendix S2: Holling’s equations as applied to habitat selection. Appendix S4: Shapes of the functional response curves for males and females in June and December. Appendix S5: Overview tables of the optimal models describing the three Holling types for males and females in June and December. Appendix S6: Overview of the effect of varying Holling’s type II parameters *a* and *b* on the functional response curve, linking the proportion of availability of a habitat with the proportional use.

**Additional file 2**: Appendix S3: Simulation study.

**Additional file 3**. Vignette to R package FunResp.

## Data Availability

The raw data used in this study are accessible via the eurodeer database www.eurodeer.org.
