# Supplementary material for: Holling meets habitat selection: functional response of large herbivores revisited
Source: Mov Ecol. 2021 Sep 6;9:45. doi: 10.1186/s40462-021-00282-6 (PMC8422736; doi:10.1186/s40462-021-00282-6)

# Supplementary material:
# Holling meets habitat selection - functional response of large herbivores revisited

## Appendix 1a. Roe Deer Capture and Radiotelemetry Data

The management of roe deer in the BFNP is spatially limited to the wild ungulate management zone (29% of study area), such that wildlife regulation is excluded from a core area of ∼17,000 ha (Möst *et al.*, 2015). Roe deer were captured in the winter months using box traps. The animals were not chemically immobilised during attachment of a GPS-GSM neck-collar ( GPS-GSM collars (series 3.000) from VECTRONIC Aerospace, Berlin (Germany) Weilnböck *et al.*, 2012), programmed to record the position of the deer with sampling intensities ranging from every 3 min to every 12 h. Data from the first 10 days of each survey period were removed to exclude possible effects of the capture and handling of the animals on their behaviour (Morellet *et al.*, 2009). In addition, the data of animals whose fix success rate, defined as the number of successfully stored locations divided by the number of attempts (Frair *et al.*, 2010), was below <90%. were excluded from the final analysis. The average fix success rate of the remaining animals was 97%. GPS errors were uniformly distributed across the time of day ($\chi^2 = 0.04$, $df = 22$, $p > 0.999$) and time of year ($\chi^2 = 0.1$, $df = 10$, $p > 0.999$), missing values were discarded from the analysis (n=6,138).

Before thinning, 172,507 fixes were obtained for 52 roe deer (26 males, 26 females), ranging from 136 to 17,044 fixes per individual (mean: 3,317, SD: 2,897), over a period of 14–2,081 days (mean: 484, SD: 397). The average spatial accuracy of the fixes was 10 m, with a maximum recorded error of 16.3 m(Stache, Löttker & Heurich, 2012).

Spatial autorcorrelation was analysed using variograms (Fleming *et al.*, 2014). For the monthly habitat selection, it is assumed that successive locations are independent at the scale of the home range, i.e. that the animal might have crossed the home range between successive steps. In the variogram, this condition is found at the time interval between successive steps where the squared displacement distance (approximately) levels off. Variograms were calculated using the package ctmm (Fleming & Calabrese, 2015) and visually inspected the variograms. In our data this interval was approximately 25 h. Only data from individuals with $> 70$ recordings were included. Per month, only the individuals with at least 10 recordings were taken into account. Thus, the final analysis consisted of 15,267 locations of 17 females and 19 males.

**Appendix 1b. Model selection & model fit**

Nineteen different models $f_i(\boldsymbol{x})$ that estimated the effects of the above mentioned variables on the odds that roe deer select habitat type i over K, were estimated for each habitat type $i = 1, \ldots, K$. To take into account the problem of overfitting, the prediction performance of all models was measured by applying cross-validation, which is finding the model $f_i(\boldsymbol{x})$ that can best predict the choice behaviour of the animals when choosing between habitat $i$ and $K$ at time $m$. Hence, models for habitats that are selected differently over time, compared to the baseline category, will in general obtain a better prediction performance. Cross-validation was applied by splitting the data into ten subgroups, ensuring that a) the data of one individual were evenly spread over all ten subgroups and b) within groups, data for all times of the year and day were available (Wiens *et al.*, 2008).
As the prediction involved a probability and the observed variable was binary, a receiver operating characteristic (ROC) curve was used for the evaluation (Agresti, 2002; Boyce *et al.*, 2002). The area under the curve (AUC) values were calculated for each testing group using the package *pROC* (Robin *et al.*, 2011), averaged and then used to identify the models with the highest predictive power. Results are shown in Figure S3.

All analyses were performed in the statistical software **R** (R Core Team, 2017) using the packages *mgcv* (Wood, 2006) for GAMMs and *adehabitatLT* for home range calculations (Calenge, 2006).

# References


Agresti, A. (2002) *Categorical Data Analysis.* John Wiley & Sons, Inc., Hoboken, New Jersey, 2nd edn.

Boyce, M., Vernier, P., Nielsen, S. & Schmiegelow, F. (2002) Evaluating resource selection functions. *Ecological Modelling*, 157, 281–300.

Calenge, C. (2006) *The package adehabitat for the R software: tool for the analysis of space and habitat use by animals.*

Fleming, C.H. & Calabrese, J.M. (2015) *ctmm: Continuous-time movement modeling.* R package version 0.2.1.

Fleming, C.H., Calabrese, J.M., Mueller, T., Olson, K.A., Leimgruber, P. & Fagan, W.F. (2014) From fine-scale foraging to home ranges: A semivariance approach to identifying movement modes across spatiotemporal scales. *The American Naturalist*, 183, E154–E167.

Frair, J.L., Fieberg, J., Hebblewhite, M., Cagnacci, F., DeCesare, N.J. & Pedrotti, L. (2010) Resolving issues of imprecise and habitat-biased locations in ecological analyses using GPS telemetry data. *Philosophical Transaction of the Royal Society B-Biological Sciences*, 365, 2187–2200.

Morellet, N., Verheyden, H., Angibault, J.M., Cargnelutti, B., Lourtet, B. & Hewison, M.A.J. (2009) The effect of capture on ranging behaviour and activity of the European roe deer *Capreolus capreolus*. *Wildlife Biology*, 15, 278–287.

Möst, L., Hothorn, T., Müller, J. & Heurich, M. (2015) Creating a landscape of management: Unintended effects on the variation of browsing pressure in a national park. *Forest Ecology and Management*, 338, 46 – 56.

R Core Team (2017) *R: A Language and Environment for Statistical Computing*. R Foundation for Statistical Computing, Vienna, Austria.
URL `https://www.R-project.org/`

Robin, X., Turck, N., Hainard, A., Tiberti, N., Lisacek, F., Sanchez, J.C. & Müller, M. (2011) pROC: an open-source package for R and S+ to analyze and compare ROC curves. *BMC Bioinformatics*, 12, 77.

Stache, A., Löttker, P. & Heurich, M. (2012) Red deer telemetry: Dependency of the position acquisition rate and accuracy of GPS collars on the structure of a temperate forest dominated by European beech (*Fagus sylvatica*) and Norway spruce (*Picea abies*). *Silva Gabreta*, 18(1), 35–41.

Weilnböck, G., Wöhr, C., Erhard, M., Menges, V., Scheipl, F., Möst, L., Palme, R. & Heurich, M. (2012) Zur Stressbelastung des Rehwilds (*Capreolus capreolus*) beim Fang mit der Kastenfalle. KTBL-Schrift 496: Aktuelle Arbeiten zur artgemäßen Tierhaltung 2012. *44. Internationale Arbeitstagung Angewandte Ethologie bei Nutztieren der DVG*, pp. 22–31.

Wiens, T.S., Dale, B.C., Boyce, M.S. & Kershaw, G.P. (2008) Three way k-fold cross-validation of resource selection functions. *Ecological Modelling*, 212, 244–255.

Wood, S. (2006) *Generalized additive models: An introduction with R*. Chapman and Hall/CRC.

**Figure 1.** AUC values display the goodness of model prediction of the choice behaviour of the animals when choosing between habitat $i$ and $K$ for $i = 1, \ldots, 11$ habitat types. AUC-values are in ascending order of the sum of AUC-values (over habitats). The greater the AUC value the better is the prediction performance of the model. Model components integrated in the model are shown in the table on the bottom of the figure. All models included the variable id (for individual) and year as random effects. Abbrevations: rel.avail, relative avialability of habitat type; s, smooth term, for hour and month it is a cyclic smooth function; te, cyclic tensor product smooth term; by, a replicate of the smooth is produced for each factor level of sex or season or interaction of both, respectively.

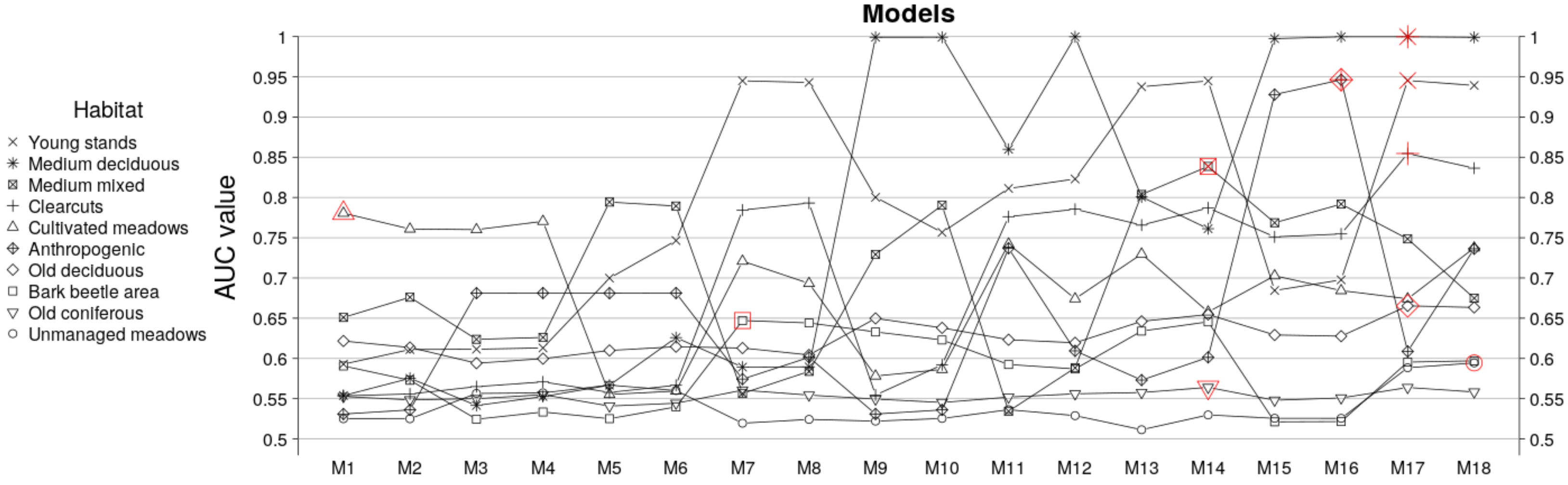


| Model components | M1 | M2 | M3 | M4 | M5 | M6 | M7 | M8 | M9 | M10 | M11 | M12 | M13 | M14 | M15 | M16 | M17 | M18 | |
|---|---|---|---|---|---|---|---|---|---|---|---|---|---|---|---|---|---|---|---|
| id (random effect) | × | × | × | × | × | × | × | × | × | × | × | × | × | × | × | × | × | × | id (random effect) |
| year (random effect) | × | × | × | × | × | × | × | × | × | × | × | × | × | × | × | × | × | × | year (random effect) |
| sex | × | × | × | × | × | × | × | | × | × | × | | × | | × | | | × | sex |
| season | | | × | × | × | × | × | × | | | | | × | × | | | | | season |
| te(month, hour) | | × | × | | × | | | × | | × | | × | | × | | × | × | | te(month, hour) |
| te(month, hour, by = sex) | × | | | × | | × | × | | × | | × | | × | | × | | | × | te(month, hour, by = sex) |
| offset(log(rel.avail)) | × | × | × | × | × | × | × | × | × | × | × | × | × | × | × | × | × | × | offset(log(rel.avail)) |
| rel.avail | × | × | × | × | | | × | × | | | × | × | | | | | | | rel.avail |
| rel.avail*sex | × | × | × | × | | | | | | | | | | | | | | | rel.avail*sex |
| rel.avail*season | | | | | | | × | × | | | | | | | | | | | rel.avail*season |
| rel.avail*sex*season | | | × | × | | | | | | | | | | | | | | | rel.avail*sex*season |
| s(rel.avail) | | | | | | | | | | | | | | | | | × | × | s(rel.avail) |
| s(rel.avail, by = sex) | | | | | | | | | × | × | | | | | | | | | s(rel.avail, by = sex) |
| s(rel.avail, by = season) | | | | | | | | | | | | | × | × | | | | | s(rel.avail, by = season) |
| s(rel.avail, by = season*sex) | | | | | × | × | | | | | | | | | | | | | s(rel.avail, by = season*sex) |
| mean AUC | 0.595 | 0.598 | 0.601 | 0.606 | 0.61 | 0.623 | 0.651 | 0.653 | 0.655 | 0.659 | 0.676 | 0.677 | 0.696 | 0.698 | 0.706 | 0.71 | 0.724 | 0.734 | |

**Appendix S2: Holling's equation as applied to habitat selection**

**Calculating the point of switch when use equals availability for Holling type II.**

$$y(x) = \frac{ax}{b+x} \qquad \mid y(x) = x \tag{1}$$
$$x = \frac{ax}{b+x} \qquad \mid \times (b+x) \tag{2}$$
$$xb + x^2 = ax \tag{3}$$
$$0 = x^2 + bx - ax \tag{4}$$
$$0 = x^2 + (b-a)x \qquad \mid : x \quad (x_1 = 0) \tag{5}$$
$$0 = x + b - a \tag{6}$$
$$x_2 = a - b \tag{7}$$

**Calculating the point of switch when use equals availability for Holling type III.**

$$y(x) = \frac{ax^2}{b^2+x^2} \qquad \mid y(x) = x \tag{8}$$
$$x = \frac{ax^2}{b^2+x^2} \qquad \mid \times (b^2+x^2) \tag{9}$$
$$b^2x + x^3 = ax^2 \tag{10}$$
$$0 = x^3 - ax^2 + b^2x \qquad \mid : x \quad (x_1 = 0) \tag{11}$$
$$0 = x^2 - ax + b^2 \tag{12}$$
$$x_{2/3} = \frac{a}{2} \pm \sqrt{\left(\frac{a}{2}\right)^2 - b^2} \tag{13}$$

**Calculating the inflection point for Holling type III.**

First derivative

$$\begin{aligned}
\frac{dy}{dx} &= \frac{d}{dx}\frac{ax^2}{b^2+x^2} \\
&= \frac{2ax(b^2+x^2) - 2ax^3}{(b^2+2x)^2} \\
&= \frac{2ab^2x + 2ax^3 - 2ax^3}{(b^2+2x)^2} \\
&= \frac{2ab^2x}{(b^2+2x)^2}
\end{aligned}$$

Second derivative

$$\begin{aligned}
\frac{d^2y}{dx^2} &= \frac{d}{dx}\frac{2ab^2x}{(b^2+2x)^2} \\
&= \frac{2ab^2(b^2+2x)^2 - 8ab^2x^2(b^2+x^2)}{(b^2+2x)^4} \\
&= \frac{(2ab^4 + 2ab^2x^2 - 8ab^2x^2)(b^2+x^2)}{(b^2+x^2)^4} \\
&= \frac{(2ab^4 - 6ab^2x)}{(b^2+x^2)^3} = \frac{2ab^2(b^2-3x^2)}{(b^2+x^2)^3}
\end{aligned}$$

Inflection point:

$$
\begin{aligned}
\frac{d^2y}{dx^2} &\overset{!}{=} 0 \\
0 &= \frac{2ab^2(b^2 - 3x^2)}{(b^2 + x^2)^3} \\
0 &= 2ab^2(b^2 - 3x^2) \\
0 &= b^2 - 3x^2 \\
x^2 &= \frac{b^2}{3} \\
x_1 = b/\sqrt{3} \quad & and \quad x_2 = -b/\sqrt{3}
\end{aligned}
$$

## Appendix S4: Shapes of functional response curves

**Figure S4.1. Shapes of functional response curves.** Shapes of functional response curves for all habitats in **June** for 17 **females** roe deer during night (red dashed line) and day (green dotdashed line) recorded in the National Park Bavarian Forest, Germany from 2005 to 2012. Black lines in the background of the coloured curves are the estimates based on multicategory logit models. Grey line indicates proportionality between use and availabilty with factor 1.

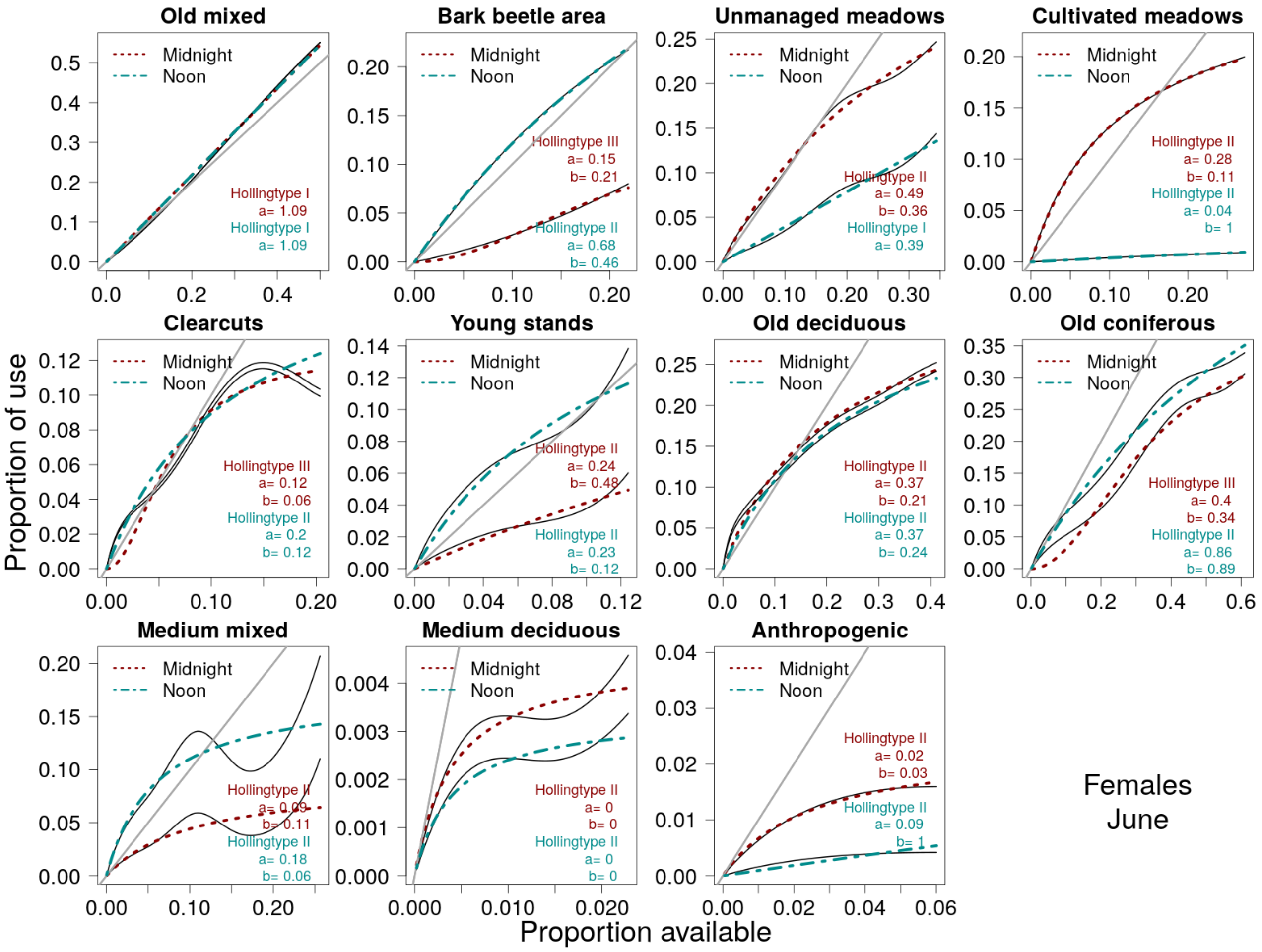

**Figure S4.2. Shapes of functional response curves.** Shapes of functional response curves based on Holling's types I, II or III for all habitats in **December** for 19 **males** roe deer during night (red dashed line) and day (green dotdashed line) recorded in the National Park Bavarian Forest, Germany from 2005 to 2012. Black lines in the background of the coloured curves are the estimates based on multicategory logit models. Grey line indicates proportionality between use and availabilty with factor 1.

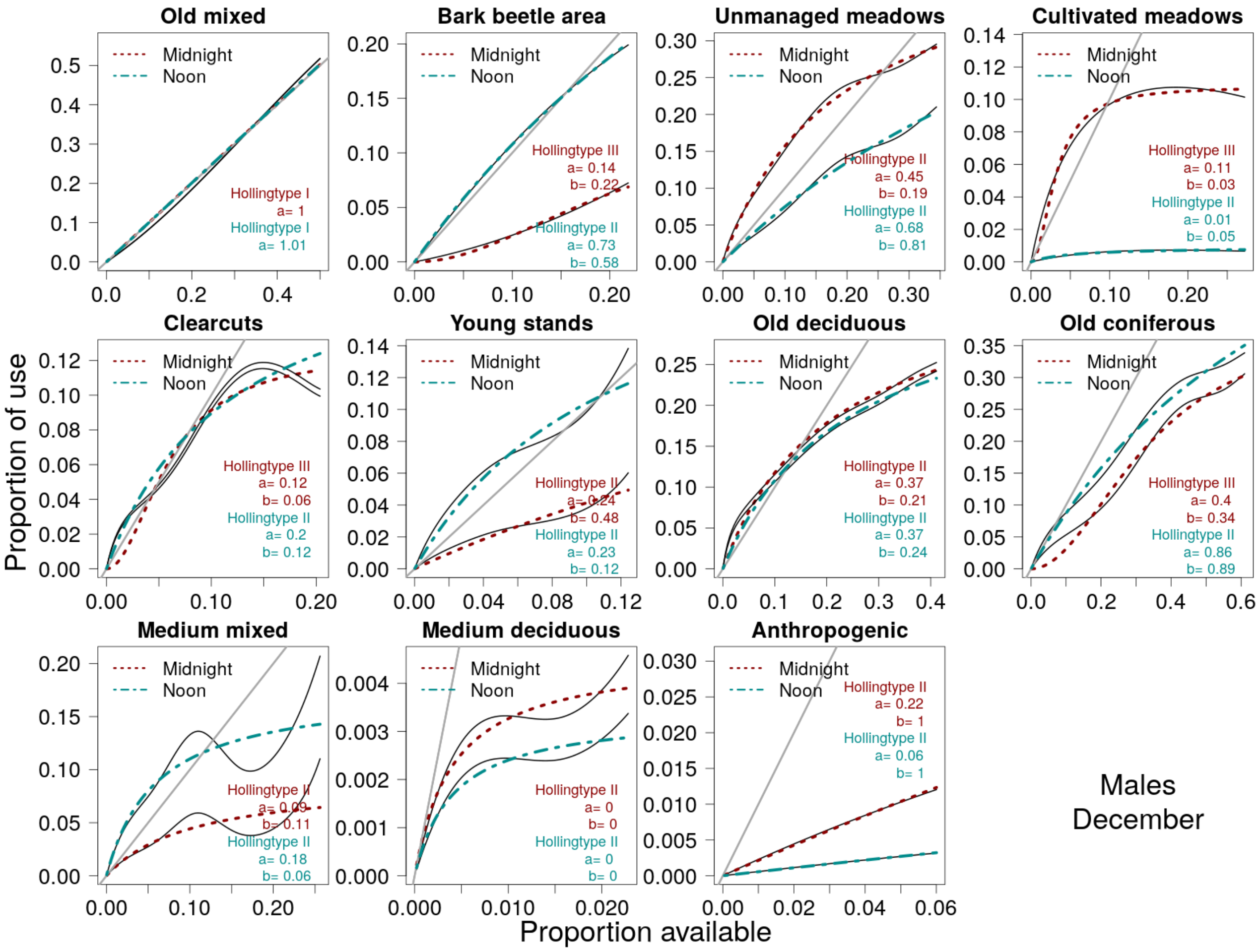

**Figure S4.3. Shapes of functional response curves.** Shapes of functional response curves for all habitats in **December** for 17 **females** roe deer during night (red dashed line) and day (green dotdashed line) recorded in the National Park Bavarian Forest, Germany from 2005 to 2012. Black lines in the background of the coloured curves are the estimates based on multicategory logit models. Grey line indicates proportionality between use and availabilty with factor 1.

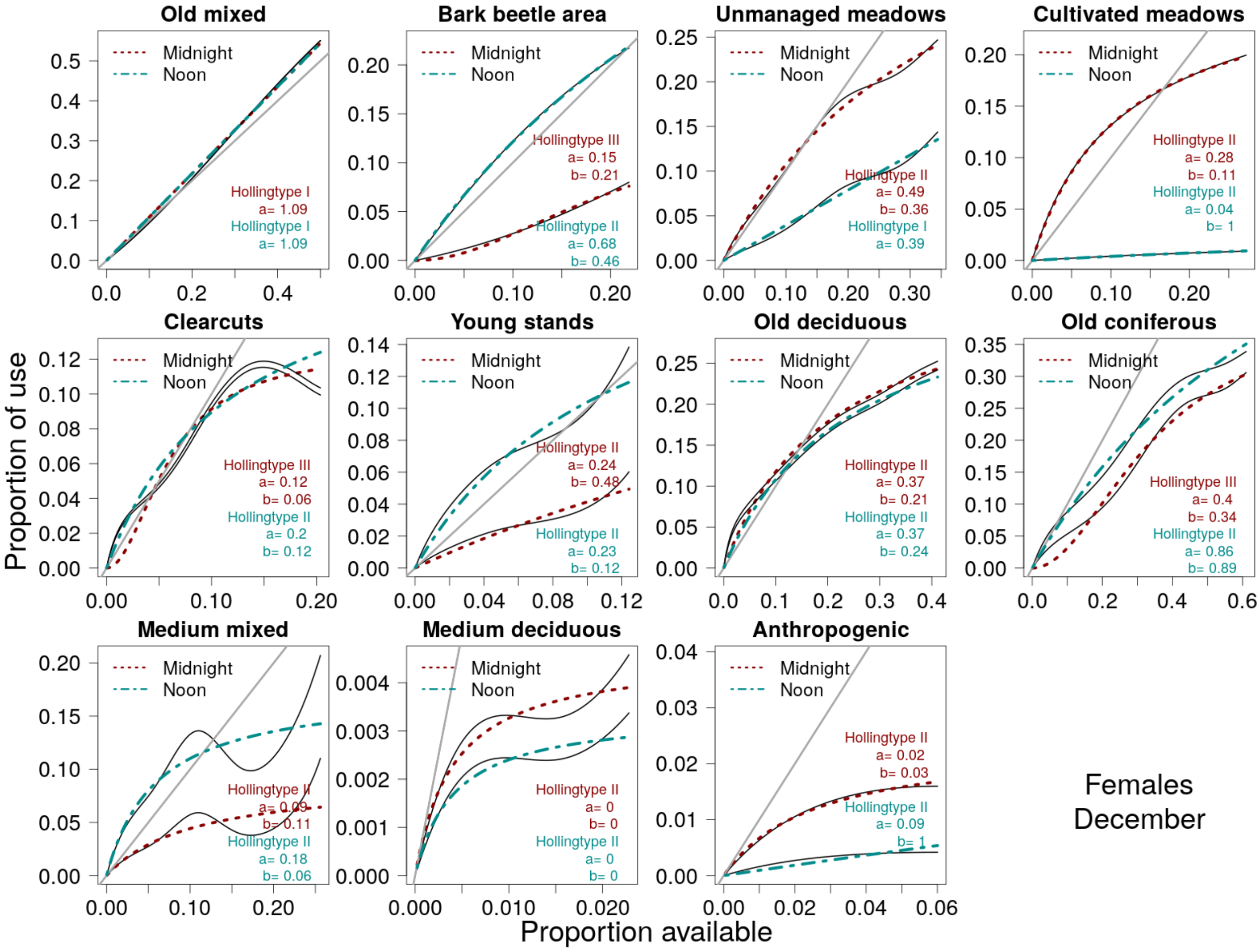

**Appendix S5: Overview of optimal models describing Holling types**

Given Holling's equations for type I: $h_I(x) = ax$, where x is the availability of a habitat, a value between 0 and 1; for type II: $h_{II}(x) = \frac{ax}{b+x}$ and for type III: $h_{III}(x) = \frac{ax^2}{b^2+x^2}$ and the estimated curves for functional response the optimal values for $a$ and $b$ are evaluated that minimizes the distance between the estimated curves and one of the Holling functions. The optimal values are listed in the following tables for different times of the year (month: June or December) and day (noon or midnight) and for males and females. Furthermore, the fraction $\frac{a}{b}$ indicates the selection strength independent of availability of a habitat: the greater the value the greater the general use. $x^*$ for Holling type II is the availability at which use equals availability, hence the value of relative availability at which no selection occurs, which is the tipping point when selection switches to avoidance of a habitat.

| Habitat | Sex | Month | Hour | type | a | b | $a/b$ | $x^*$ |
|---|---|---|---|---|---|---|---|---|
| Old mixed | m | 12 | 0 | I | 1.00 | | | 0 |
| | | | 12 | I | 1.01 | | | 0 |
| Bark beetle area | m | 12 | 0 | III | 0.14 | 0.22 | 0.63 | 0 |
| | | | 12 | II | 0.73 | 0.58 | 1.26 | 0.15 |
| Unmanaged meadows | m | 12 | 0 | II | 0.45 | 0.19 | 2.42 | 0.26 |
| | | | 12 | II | 0.68 | 0.81 | 0.84 | 0 |
| Cultivated meadows | m | 12 | 0 | III | 0.11 | 0.03 | 3.36 | 0 |
| | | | 12 | II | 0.01 | 0.05 | 0.18 | 0 |
| Clearcuts | m | 12 | 0 | III | 0.12 | 0.06 | 2.07 | 0 |
| | | | 12 | II | 0.20 | 0.12 | 1.64 | 0.08 |
| Young stands | m | 12 | 0 | II | 0.24 | 0.48 | 0.50 | 0 |
| | | | 12 | II | 0.23 | 0.12 | 1.90 | 0.11 |
| Old deciduous | m | 12 | 0 | II | 0.37 | 0.21 | 1.72 | 0.16 |
| | | | 12 | II | 0.37 | 0.24 | 1.52 | 0.13 |
| Old coniferous | m | 12 | 0 | III | 0.40 | 0.34 | 1.17 | 0 |
| | | | 12 | II | 0.86 | 0.89 | 0.97 | 0 |
| Medium mixed | m | 12 | 0 | II | 0.09 | 0.11 | 0.86 | 0 |
| | | | 12 | II | 0.18 | 0.06 | 2.93 | 0.12 |
| Medium deciduous | m | 12 | 0 | II | 0.00 | 0.00 | 1.13 | 0 |
| | | | 12 | II | 0.00 | 0.00 | 0.83 | 0 |
| Anthropogenic | m | 12 | 0 | II | 0.22 | 1.00 | 0.22 | 0 |
| | | | 12 | II | 0.06 | 1.00 | 0.06 | 0 |

| Habitat | Sex | Month | Hour | type | a | b | $a/b$ | $x^*$ |
|---|---|---|---|---|---|---|---|---|
| Old mixed | f | 6 | 0 | I | 0.74 | | | 0 |
| | | | 12 | I | 0.80 | | | 0 |
| Bark beetle area | f | 6 | 0 | I | 0.48 | | | 0 |
| | | | 12 | II | 0.29 | 0.08 | 3.61 | 0.21 |
| Unmanaged meadows | f | 6 | 0 | II | 0.25 | 0.02 | 13.13 | 0.23 |
| | | | 12 | II | 0.34 | 0.30 | 1.14 | 0.04 |
| Cultivated meadows | f | 6 | 0 | II | 0.23 | 0.02 | 13.64 | 0.22 |
| | | | 12 | II | 0.19 | 0.13 | 1.47 | 0.06 |
| Clearcuts | f | 6 | 0 | II | 0.18 | 0.07 | 2.51 | 0.11 |
| | | | 12 | II | 0.20 | 0.09 | 2.35 | 0.12 |
| Young stands | f | 6 | 0 | II | 0.16 | 0.12 | 1.35 | 0.04 |
| | | | 12 | II | 0.20 | 0.06 | 3.52 | 0.15 |
| Old deciduous | f | 6 | 0 | II | 0.24 | 0.18 | 1.32 | 0.06 |
| | | | 12 | II | 0.30 | 0.21 | 1.45 | 0.09 |
| Old coniferous | f | 6 | 0 | I | 0.37 | | | 0 |
| | | | 12 | I | 0.46 | | | 0 |
| Medium mixed | f | 6 | 0 | III | 0.19 | 0.21 | 0.90 | 0 |
| | | | 12 | II | 0.82 | 0.72 | 1.14 | 0.10 |
| Medium deciduous | f | 6 | 0 | II | 0.13 | 1.00 | 0.13 | 0 |
| | | | 12 | II | 0.00 | 0.00 | 0.87 | 0 |
| Anthropogenic | f | 6 | 0 | III | 0.02 | 0.01 | 2.31 | 0 |
| | | | 12 | II | 0.04 | 1.00 | 0.04 | 0 |

| Habitat | Sex | Month | Hour | type | a | b | $a/b$ | $x^*$ |
|---|---|---|---|---|---|---|---|---|
| Old mixed | f | 12 | 0 | I | 1.09 | | | 0 |
| | | | 12 | I | 1.09 | | | 0 |
| Bark beetle area | f | 12 | 0 | III | 0.15 | 0.21 | 0.69 | 0 |
| | | | 12 | II | 0.68 | 0.46 | 1.48 | 0.22 |
| Unmanaged meadows | f | 12 | 0 | II | 0.49 | 0.36 | 1.37 | 0.13 |
| | | | 12 | I | 0.39 | | | 0 |
| Cultivated meadows | f | 12 | 0 | II | 0.28 | 0.11 | 2.48 | 0.17 |
| | | | 12 | II | 0.04 | 1.00 | 0.04 | 0 |
| Clearcuts | f | 12 | 0 | III | 0.12 | 0.06 | 2.07 | 0 |
| | | | 12 | II | 0.20 | 0.12 | 1.64 | 0.08 |
| Young stands | f | 12 | 0 | II | 0.24 | 0.48 | 0.50 | 0 |
| | | | 12 | II | 0.23 | 0.12 | 1.90 | 0.11 |
| Old deciduous | f | 12 | 0 | II | 0.37 | 0.21 | 1.72 | 0.16 |
| | | | 12 | II | 0.37 | 0.24 | 1.52 | 0.13 |
| Old coniferous | f | 12 | 0 | III | 0.40 | 0.34 | 1.17 | 0 |
| | | | 12 | II | 0.86 | 0.89 | 0.97 | 0 |
| Medium mixed | f | 12 | 0 | II | 0.09 | 0.11 | 0.86 | 0 |
| | | | 12 | II | 0.18 | 0.06 | 2.93 | 0.12 |
| Medium deciduous | f | 12 | 0 | II | 0.00 | 0.00 | 1.13 | 0 |
| | | | 12 | II | 0.00 | 0.00 | 0.83 | 0 |
| Anthropogenic | f | 12 | 0 | II | 0.02 | 0.03 | 0.93 | 0 |
| | | | 12 | II | 0.09 | 1.00 | 0.09 | 0 |

Figure S1: **Appendix S6.** Overview of the effect of varying parameters $a$ and $b$ of the Holling's type II on the functional response curve, linking the proportion of availability of a habitat with the proportion of its use.

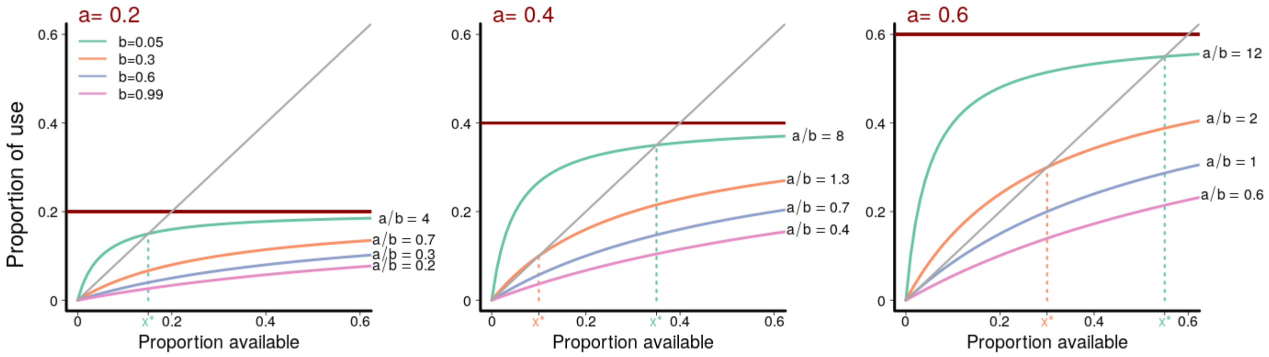

Supplement: Supplementary file 1 — Addtional file 1: Appendix S1a: Roe Deer Capture and Radiotelemetry Data. Appendix S1b: Model selection and model fit. Appendix S2: Holling’s equations as applied to habitat selection. Appendix S4: Shapes of the functional response curves for males and females in June and December. Appendix S5: Overview tables of the optimal models describing the three Holling types for males and females in June and December. Appendix S6: Overview of the effect of varying Holling’s type II parameters a and b on the functional response curve, linking the proportion of availability of a habitat with the proportional use. [file 40462_2021_282_MOESM1_ESM.pdf]
