# Supplementary material for: Holling meets habitat selection: functional response of large herbivores revisited
Source: Mov Ecol. 2021 Sep 6;9:45. doi: 10.1186/s40462-021-00282-6 (PMC8422736; doi:10.1186/s40462-021-00282-6)
Supplement: Supplementary file 2 — Additional file 2: Appendix S3: Simulation study. [file 40462_2021_282_MOESM2_ESM.pdf]

# Appendix S3 - Simulation study: Holling meets habitat selection

Claudia Dupke, Anne Peters, Nicolas Morellet and Marco Heurich

13 5 2021

## Aim

Can Holling's types really be used to describe habitat use as a function of their availability? In this study we aim to provide an methodological overview how Holling's types and habitat selection analysis can be linked.

## Approach

For this purpose, data is first simulated that shows the choice behaviour of individuals that have different home ranges with an individual composition of the three types of land cover: forest, openland and regeneration area. Choice probability of a land cover types varies over the course of the day and is affected by the availability of land cover type, whereby the probability that a specific land cover type is chosen by an animal increases with increasing availability in its home range.

Based on the simulated choices of animals during the day and over several days, the proportional use of each land cover type is related to its availability in an animal's home range. To these simulated curves Holling's types were fitted.

## Process

**Preparation of the environment** In this section the number of individuals, the number of patches, the number of days are set to be used for the simulation. For each individual the home range composition is simulated to derive the availabilities of the three landcover type in each home range.

**Definition of the selection probability** The choice probability $\pi_i$ is the probability that a land cover type $i$ is chosen out of a choice set $i = \{1, 2, 3\}$. As we assume that the animal has to choose from exactly three land cover types the probabilities must sum to 1: $\pi_1 + \pi_2 + \pi_3 = 1$. In such a discrete choice framework selection probabilities can be denoted by using baseline logits. The logits or the log odds ratios from baseline logit models contrast the probability of selecting a specific land cover type with the probability of selecting a reference land cover type (which is the baseline). Here, the assumption is that the odds ratios vary over the time of day and with respect to the availability of a land cover type. The user can play around with different values. We first assume a directly proportional relationship between selection probability and availability, which means that selection probability increases with availability by a factor of 1.

The odds ratios are defined for two land cover type (with respect to the reference type) and then used to calculate choice probabilities for the landcover where the mutlinomial logit ensures that $\pi_1 + \pi_2 + \pi_3 = 1$.

In our special case the choice probabilities are equivalent to the probabilities of use since the choice set is identical with the set of available land cover types. Furthermore, the probability of use is identical with the proportional use as we focus on discrete land cover categories.

**Fitting Holling types (1st time)** The choice probabilities are visualised over the time of day and over the range of observed availability, revealing the multinomial structure of the data. The curves choice probability vs. rel. availability were used to fit Holling types. These are the parameters that are used to validate the methodology that is presented in the accompanying article.

**Simulating habitat selection** Based on the choice probabilities (which varied for time of day and between individuals), land cover types were randomly chosen by each individual over the simulation period.

**Estimating choice probability based on simulated data** The simulated data was used to estimate two binary logits models to obtain the odds ratios for selecting forest vs. regeneration area and openland vs. regeneration area. Given the odds ratios choice probabilities (or proportional use) could be estimated over the time of day or over the range of observed availabilities.

**Fitting Holling types (2nd time)** The Holling types were fitted to the latter curves and the estimated values were compared with the earlier estimated values of the Holling curves.

**Checking the model performance** At the end we summarize our findings.

## Start of the simulation

### Preparation of the environment

Prepare the R environment and set the number of individuals, number of patches within a home range and number of days you want to simulate. We basically simulate habitat selection of N individuals which have the choice between three types of landcover, e.g. openland, forest, regeneration area.

```
set.seed(1947)

library(knitr)
library(ggplot2)
library(tidyverse)
library(scales)
library(mgcv)
library(ggpubr)

frac <- 1

#number of individuals
N <- 100
#number of patches within each home range
npatches <- 100
#number of days
ndays <- 10
```

We simulate 100 individuals with homeranges that consists of 100 patches with varying availabilites of the three landcover types. In the first step, for each individual the number of patches that belongs to forest or open landcover are simulated from a uniform distribution, where we set the upper limit of availability (upper1, upper2). Finally, we show the simulated distribution of availabilities in each home range.

```
upper1 <- 0.5
upper2 <- 0.5
avail1 <- sample(1:(npatches*upper1), N, replace = TRUE, prob = rep(1, npatches*upper1))
names(avail1) <- 1:N
avail2 <- sample(1:(npatches*upper2), N, replace = TRUE, prob = rep(1, npatches*upper2))
names(avail2) <- 1:N
avail3 <- npatches - avail1 - avail2
names(avail3) <- 1:N

Avail <- cbind(ID = 1:N, forest = avail1, openland = avail2, reg.area = avail3)
#unlist( apply(Avail,1, sum))
Avail2 <- data.frame(ID = factor(1:N), habitat = rep(c("forest","openland", "reg.area"),
                                                    each = N),
```

```
                              availibility = as.numeric(c(avail1,avail2, avail3)))

ggplot(Avail2, aes(x= ID, y= availibility, fill =habitat))+
        geom_bar(position="stack",stat="identity")
```

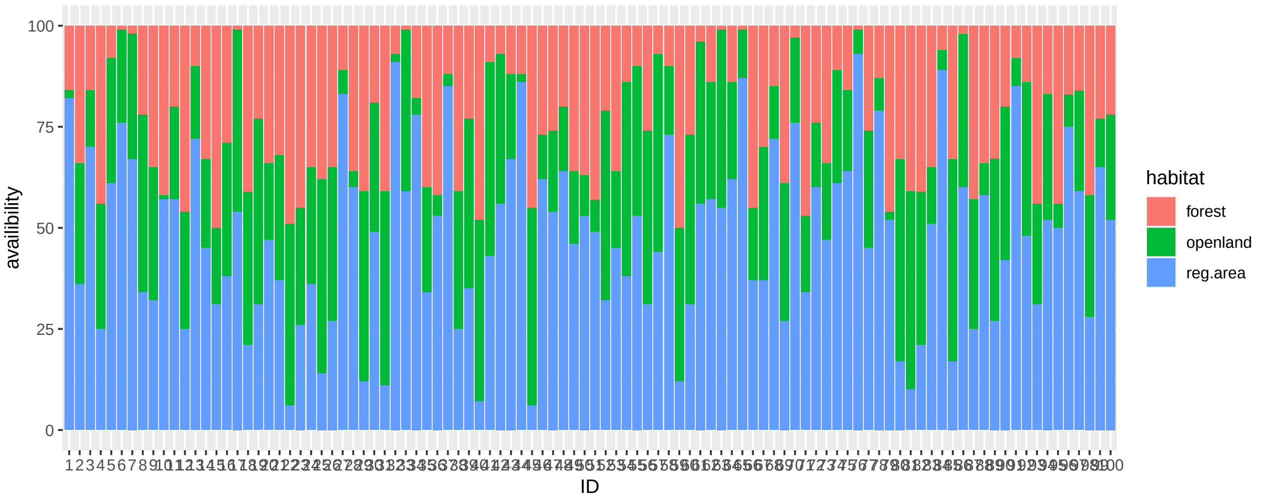


### Definition of the selection probability

We now want to define the choice probability of a land cover type. We assume that the probability of choosing a land cover type varies over the time of day (*tod*), namely that forest is selected with a higher probability during the day and openland with a lower probability during the day compared to regeneration area. Therefore, we define two cyclic functions *Pr(Forest = 1| tod)* and *Pr(Openland = 1| tod)* over the time of day that provide the odds ratios over the time of day, which is the fraction of the probability of selecting forest (/openland) versus the probability of regeneration area. The probabilities of choosing either forest or openland or regeneration area are derived via the multinomial logit link. The probability of choosing regeneration area is the complementary probability of choosing forest or openland, namely *Pr(regeneration = 1| tod) = 1 - Pr(Forest = 1| tod) - Pr(Openland = 1| tod)*.

```
TOD <- seq(0,24)

Df <- data.frame(ID = rep(1:N,  each = length(TOD)*ndays),
                 Nday = rep(rep(1:ndays, each = length(TOD)), times = N),
                 tod = rep(TOD, times = N*ndays))
Df$avail1 <- Avail[Df$ID,"forest"]
Df$avail2 <- Avail[Df$ID,"openland"]
Df$rel.avail1 <- Avail[Df$ID,"forest"]/npatches
Df$rel.avail2 <- Avail[Df$ID,"openland"]/npatches

#log odds ratios
get_logOddsRatio <- function(avail, x, t){
     x[1] + 1 * log(avail+0.001) + x[2]*log(avail+0.001) + x[3]*cos(pi*t/12)
}

#compute log odds ratios over time of day for different valuies of availability
Df$logOR1 <- unlist(sapply(1:nrow(Df), function(i)
                get_logOddsRatio(Df[i,"rel.avail1"], c(0.2,0,-0.5), Df[i,"tod"])))
Df$logOR2 <- unlist(sapply(1:nrow(Df), function(i)
                get_logOddsRatio(Df[i,"rel.avail2"], c(0.5,0,2), Df[i,"tod"])))
Df$OR1 <- exp(Df$logOR1)
```

```r
Df$OR2 <- exp(Df$logOR2)

Df$p1 <- Df$OR1/(1+Df$OR1 + Df$OR2)
Df$p2 <- Df$OR2/(1+Df$OR1 + Df$OR2)
Df$p3 <- 1- Df$p1 - Df$p2
```

We can plot the variation of choice probability over the course of the day for each individual (for one individual the relative avialability within its home range is constant over time).

```r
G1 <- ggplot(Df, aes(x = tod, y = p1, group = ID, col = rel.avail1)) +  geom_line() +
                  ylim(0,1) + xlim(0,24) + ggtitle("Choice probability of forest")
G2 <- ggplot(Df, aes(x = tod, y = p2, group = ID, col = rel.avail2)) +  geom_line() +
                  ylim(0,1)+ xlim(0,24)+ ggtitle("Choice probability of openland")
G3 <- ggplot(Df, aes(x = tod, y = p3, group = ID, col = 1-rel.avail1-rel.avail2)) +
        geom_line() + ylim(0,1)+ xlim(0,24)+
        ggtitle("Choice probability of reg. area") +
        guides(col=guide_legend(title="rel.avail"))

ggarrange(G1,G2, G3 , ncol = 3)
```

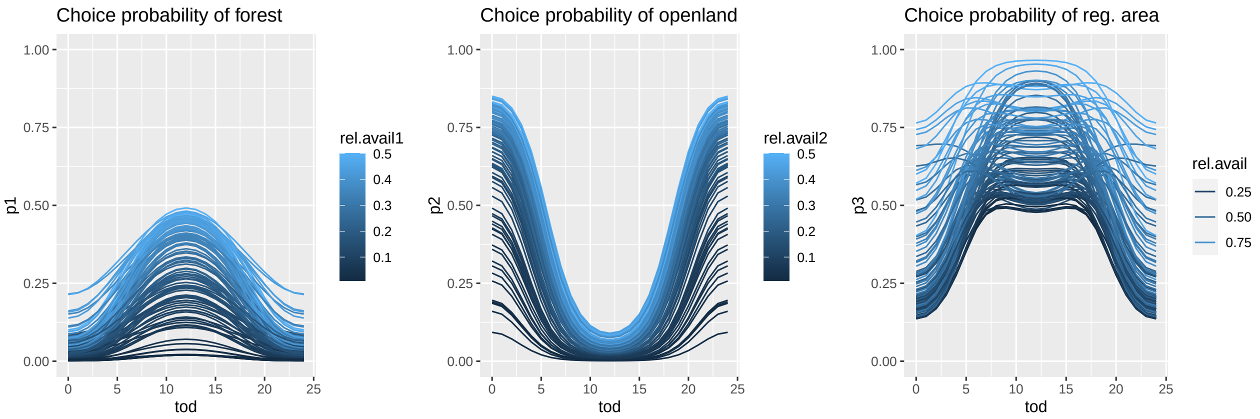


We can also plot these choice probabilities a landcover type with respect to its availabiliy in the home ranges.

```r
G1 <- ggplot(Df, aes(x = rel.avail1, y = p1, group = factor(tod), col = factor(tod))) +
          geom_point() + geom_smooth() + ylim(0,1)+
          ggtitle("Choice probability of forest") + geom_abline(intercept = 0)
G2 <- ggplot(Df, aes(x = rel.avail2, y = p2, group = as.factor(tod), col = factor(tod))) +
          geom_point() + geom_smooth() + ylim(0,1)+
          ggtitle("Choice probability of openland") + geom_abline(intercept = 0)
G3 <- ggplot(Df, aes(x = 1-rel.avail1-rel.avail2, y = p3, group = as.factor(tod),
                        col = factor(tod))) +
      geom_point() + geom_smooth() + ylim(0,1)+
      ggtitle("Choice probability of reg. area") +  geom_abline(intercept = 0)

ggarrange(G1,G2,G3, ncol = 2)
```

```
## `geom_smooth()` using method = 'gam' and formula 'y ~ s(x, bs = "cs")'
## `geom_smooth()` using method = 'gam' and formula 'y ~ s(x, bs = "cs")'
## `geom_smooth()` using method = 'gam' and formula 'y ~ s(x, bs = "cs")'
```

```
## $`1`
```

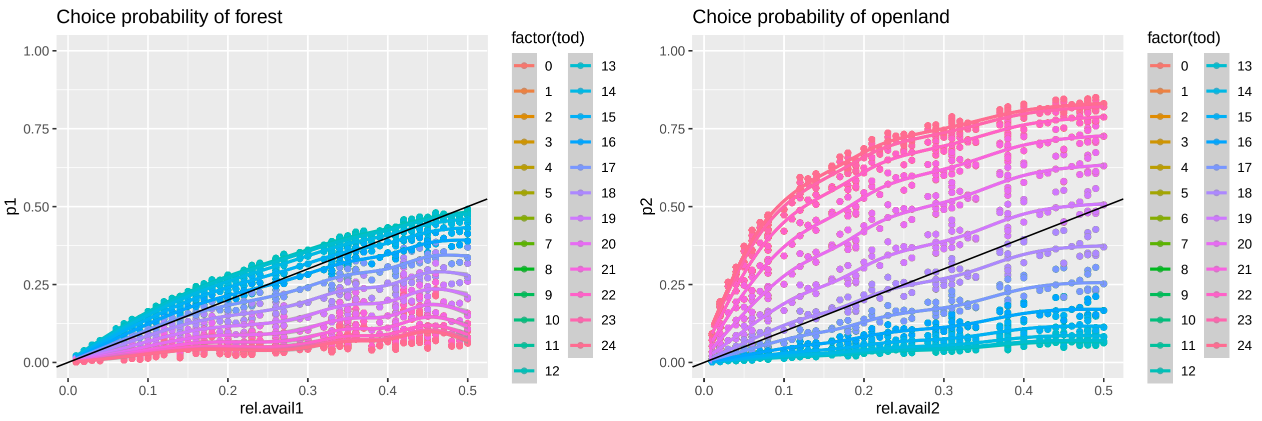


```
##
## $`2`
```

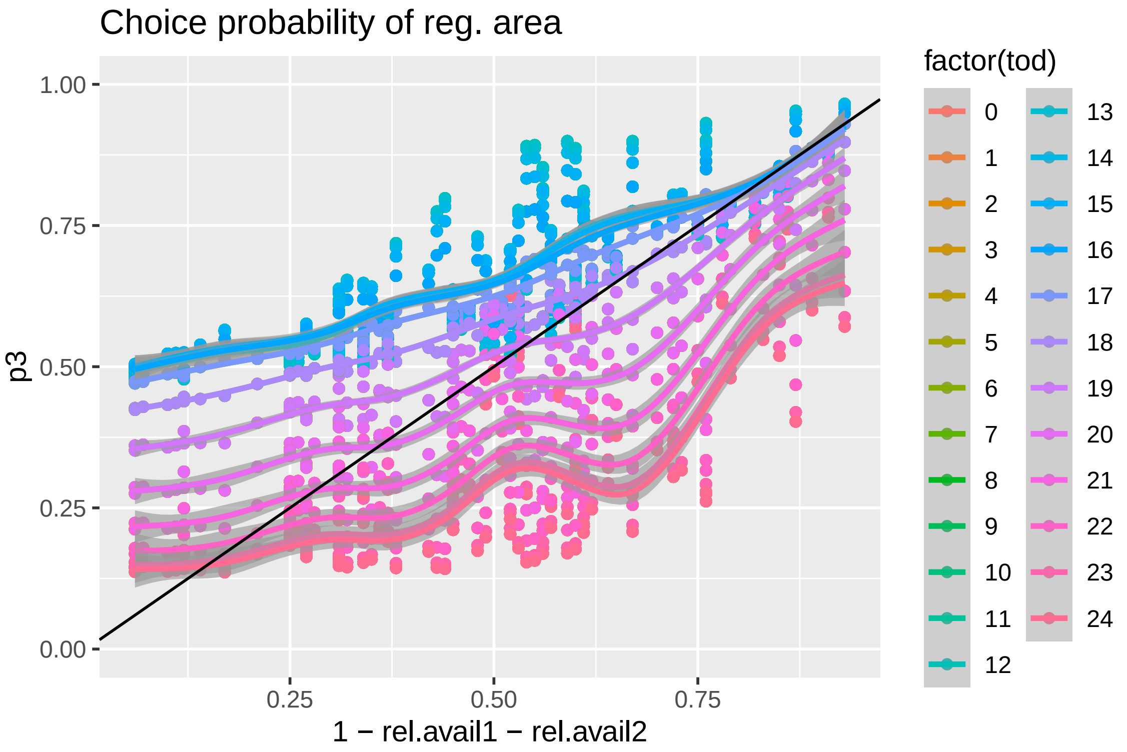


```
##
## attr(,"class")
## [1] "list"      "ggarrange"
```

### Fitting Holling types (1st time)

We see that the probability seem to be affected by time of day and the relative availability of a landcover type in a home range. We can now fit the Holling types to these probability functions. First we need to define the functions of the three Holling's types.

```
H1 <- function(x,a){
    a*x
}
H2 <- function(x,par){
      par[1]*x/(par[2]+x)
```

```
}
H3 <- function(x,par){
    par[1]*x*x/(par[2]*par[2]+x*x)
}
```

Now, we need to define the functions that we want to minimize. Basically, we want to minimize the deviation of Holling's type curves to our fitted probability curves using the least squares method.

```
min.RSSH <- function(data, func, par){
    sum((func(data[,"x"],par)-data[,"y"])^2)
}
```

Finally, we set up a function that returns the type of Holling's curves that shows the least deviation from our probability curves together with the associated parameters that describe Holling's type.

```
# optimize
getholtype <- function(ff, type = NULL){
    x <- ff[,1]
    y <- ff[,2]
    OPT <- vector("list",3)
    OPT[[1]] <- optim(par=c(1), min.RSSH, data=cbind(x,y), func = H1,
                      lower=0,upper=100, method="Brent")
    OPT[[2]] <- optim(par=c(1,0.2), min.RSSH, data=cbind(x,y), func = H2,
                      lower=0.01, upper=1, method="L-BFGS-B")
    OPT[[3]] <- optim(par=c(1,0.2), min.RSSH, data=cbind(x,y), func = H3,
                      lower=0.01, upper=1, method="L-BFGS-B")

    if(is.null(type)){
        type <- which.min(c(OPT[[1]]$value,OPT[[2]]$value,OPT[[3]]$value))
    }
    a <- OPT[[type]]$par[1]
    b <- OPT[[type]]$par[2]

    data.frame(type=type,a = round(a,5),b = round(b,5), "a/b"=round(a/(b),2))
}
```

```
HT_sim1 <- lapply(TOD ,function(t) getholtype(Df[Df$tod == t,c("rel.avail1", "p1")]))
HT_sim2 <- lapply(TOD ,function(t) getholtype(Df[Df$tod == t,c("rel.avail2", "p2")]))

frac <- mean(Avail[,"reg.area"]/npatches)
rel.avail <- seq(0, 1- frac,length = 50)

H_func <- list(H1, H2, H3)

htp_sim1 <- lapply(HT_sim1,function(X){
     H_func[[X$type]](rel.avail, c(X$a, X$b))
    })

htp_sim2 <- lapply(HT_sim2,function(X){
     H_func[[X$type]](rel.avail, c(X$a, X$b))
    })

HT_sim1 <- cbind(time_of_day = TOD,do.call(rbind,HT_sim1))
HT_sim2 <- cbind(time_of_day = TOD,do.call(rbind,HT_sim2))
DFH_sim1 <- DFH_sim2 <- expand.grid(rel.avail = rel.avail, tod = TOD)
```

```r
DFH_sim1$holling <- unlist(htp_sim1)
DFH_sim2$holling <- unlist(htp_sim2)

G1_2 <- ggplot(DFH_sim1, aes(x = rel.avail, y = holling, group = as.factor(tod),
                col = as.factor(tod))) + geom_line() +  geom_abline(intercept = 0) +
                ylim(0,1) + ggtitle("Predictions by Holling types") +
                guides(group=guide_legend(title="time of day"),
                col=guide_legend(title="time of day")) + ylab("Proportion of use")
G2_2 <- ggplot(DFH_sim2, aes(x = rel.avail, y = holling, group = as.factor(tod),
                col = as.factor(tod))) + geom_line() +  geom_abline(intercept = 0) +
                ylim(0,1) + ggtitle("Predictions by Holling types") +
                guides(group=guide_legend(title="time of day"),
                col=guide_legend(title="time of day"))+ylab("Proportion of use")

ggarrange(G1_2, G2_2)
```

```
## Warning: Removed 7 row(s) containing missing values (geom_path).
```

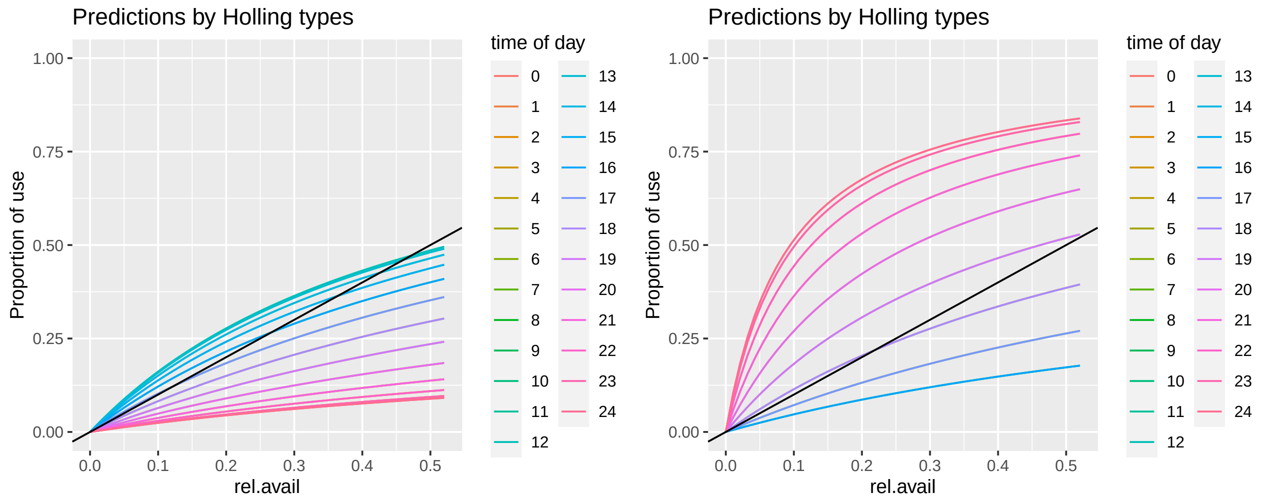


### Simulating habitat selection

Based on the choice propbability we can now simulate the selection of landcover types for each individual over the course of the day over the period of 10 days.

```r
Df$habitat <- sapply(1:nrow(Df), function(i) sample(c("forest", "openland", "reg.area"),
                    size = 1, replace = TRUE, prob = Df[i,c("p1","p2", "p3")]))

Df$forest <- ifelse(Df$habitat == "forest", 1, 0)
Df$openland <- ifelse(Df$habitat == "openland", 1, 0)
Df$reg.area <- ifelse(Df$habitat == "reg.area", 1, 0)
```

The counts of the selection of the land cover types can be summarized over time of day and over the availability of the landcover type in the home range

```r
tt <- table(Df[,c("ID", "tod", "habitat")])
df <- data.frame(ID = rep(1:N,  each = length(TOD)), tod = rep(TOD, times = N),
                habitat = rep(c("forest", "openland", "reg.area"), each = N*length(TOD)),
                counts = c(t(tt[,,"forest"]), t(tt[,,"openland"]),t(tt[,,"reg.area"])))
#availability is the value of available patches of the selected habitat
df$availability <- sapply(1:nrow(df), function(i) Avail[df$ID[i],as.character(df$habitat[i])])
```

```
#df[df$habitat=="O","availability"]   <- 1-avail[df[df$habitat=="O","ID"]]
df$rel.avail <- df$availability/npatches

G1 <- ggplot(data = df[df$habitat == "forest",], aes(x = tod, y = counts,
              group = factor(availability), col = factor(availability))) +
              geom_point() + geom_smooth() + ggtitle("Counts of forest") +
              guides(group=guide_legend(title="Available patches"),
                     col=guide_legend(title="Available patches"))
G2 <- ggplot(data = df[df$habitat == "openland",], aes(x = tod, y = counts,
              group = factor(availability), col = factor(availability))) +
              geom_point() + geom_smooth() + ggtitle("Counts of openland")+
              guides(group=guide_legend(title="Available patches"),
                     col=guide_legend(title="Available patches"))

ggarrange(G1, G2)
```

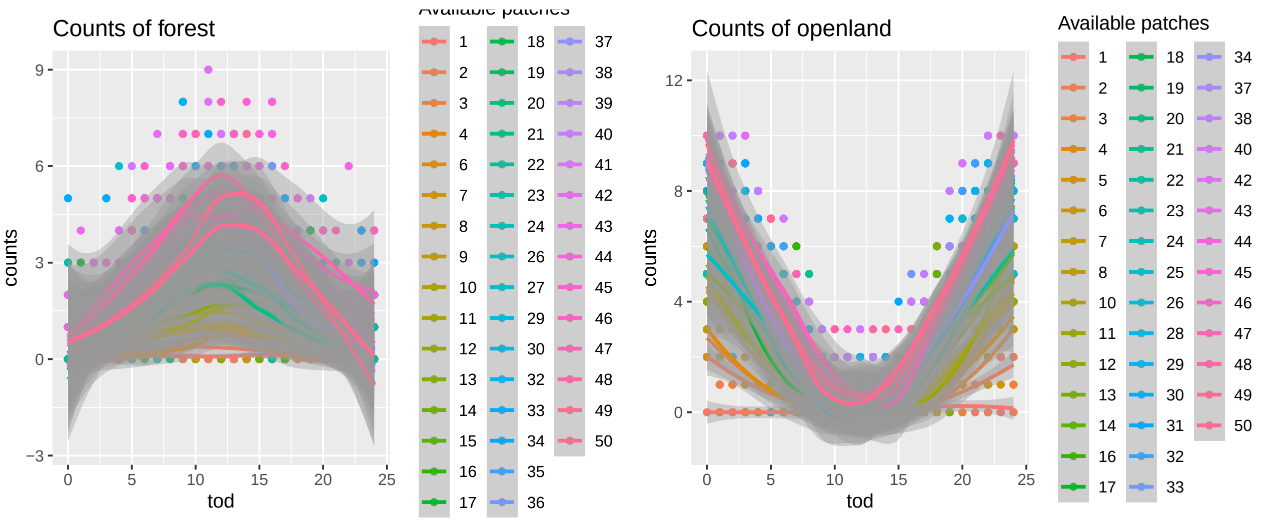


and over the availability of the landcover type in the home range:

```
G1 <- ggplot(data = df[df$habitat == "forest" & df$tod %in% c(1,3,6,9,12),],
             aes(x = rel.avail, y = counts, group = as.factor(tod) ,col = as.factor(tod))) +
            geom_point() + guides(group=guide_legend(title="time of day"),
                          col=guide_legend(title="time of day"))+   geom_smooth() +
            ylim(0,ndays)+ geom_abline(intercept = 0, slope =ndays)   +
      ggtitle("Counts of forest")

G2 <- ggplot(data = df[df$habitat == "openland" & df$tod %in% c(1,3,6,9,12),],
             aes(x = rel.avail, y = counts, group = as.factor(tod) ,col = as.factor(tod))) +
            geom_point() + guides(group=guide_legend(title="time of day"),
                          col=guide_legend(title="time of day")) + geom_smooth() +
            ylim(0,ndays)+ geom_abline(intercept = 0, slope =ndays) +
      ggtitle("Counts of openland")

ggarrange(G1,G2 )
```

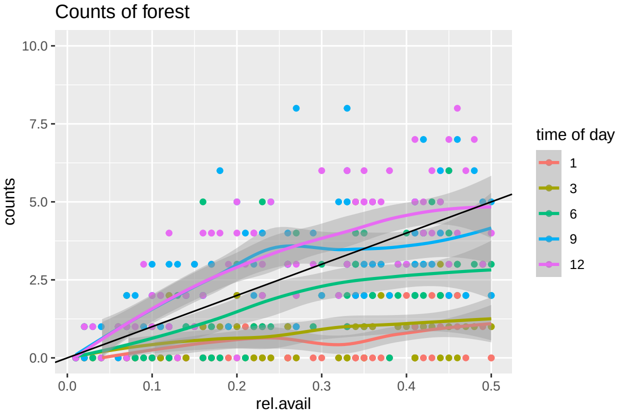


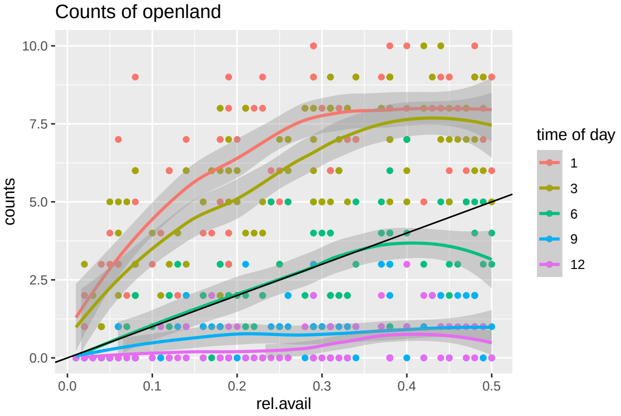


The following paragraph is to illustrate the multinomial structure of the data. For each time of day (*tod*) we can calculate the proportion of counts (or proportional use) within a time slot by averaging over all individuals. And so we can visualize the variation of proportional use over the time of day.

```
PROPS <- lapply(list("forest", "openland"), function(hh) lapply(TOD, function(t){
            DF <- subset(Df, tod == t)
            tt <- table(DF$habitat)
            prop <- tt[as.character(hh)]/sum(tt)
            prop <- ifelse(length(tt)>1, prop, ifelse(is.na(tt[as.character(hh)]),0,1))
            return(prop)
        }))

ggplot(data = data.frame(habitat = rep(factor(c("forest", "openland","reg.area")),
                        each = length(TOD)), tod = rep(TOD,3),
                        proportion = c(unlist(PROPS[[1]]),unlist(PROPS[[2]]),
                                       1- unlist(PROPS[[1]])-unlist(PROPS[[2]]))),
                aes(x = tod, y = proportion, group = habitat,col = habitat )) + geom_point() +
        geom_smooth() +ggtitle("Proportions of used habitat over time of day") + ylim(0,1) +
        xlim(0,24) + guides(col=guide_legend(title="time of day"))
```

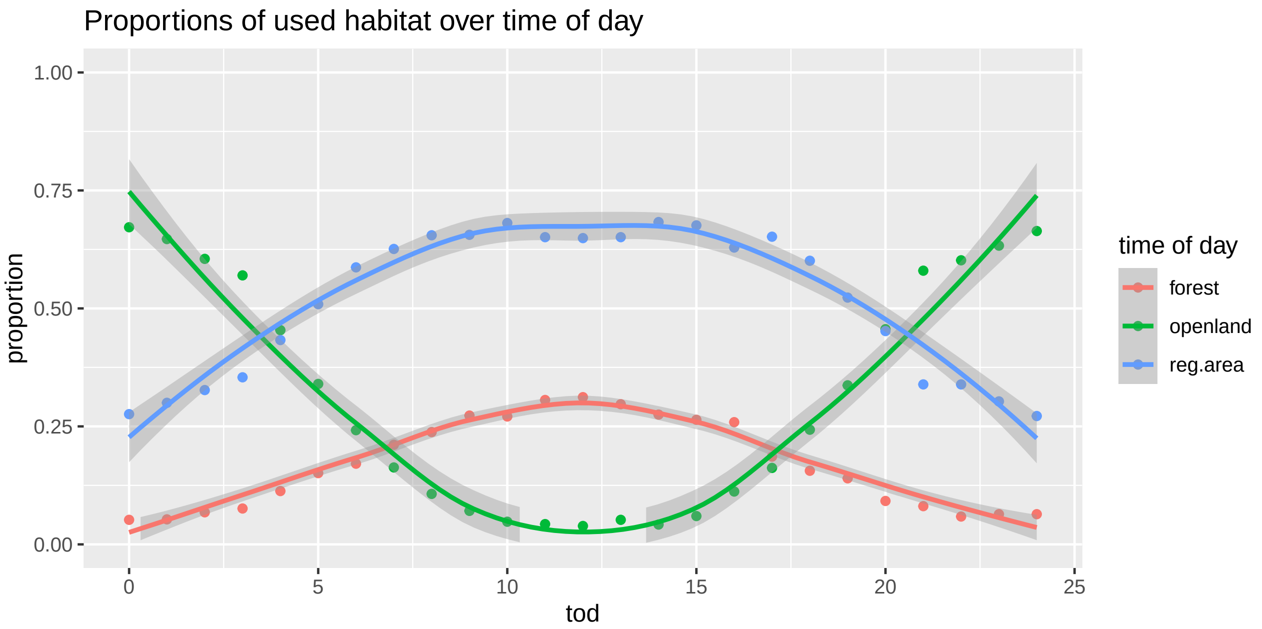

### Estimating choice probability based on simulated data

The latter calculation of proportions will only provide a meaningful overview if availability is uniformly distributed over the group of individuals, otherwise the outcome would be biased. This is usually not the case in reality. Indeed, usually there are further environmental variables that influence the habitat selection of animals. Therefore, we use a model that accounts for confounding factors. In our simulation example we did not add other effects, therefore we only include time of day *tod* and relative availability *rel.avail* as explaining factors. The offset including the log of *rel.avail* ensures that the coefficient is 1, which means that we assume the odds ratio to directly proportionally increase with the log of availability. The additional effect of rel.avail, shows whether their is a deviation from this direct proportionality.

In multicategory logit models we have to model the selection behaviour for each landcover type separately with respect to the reference type, which is in our case the regeneration area. The response is a binary variable, being 1 if the landcover type (e.g. forest) was selected and 0 if the reference type was selected. So, we need to rearrange the data set first. The subset for landscape type $i$ contained only those entries when it was selected plus the entries when the reference type was selected.

```
Df1 <- subset(Df, habitat %in% c("forest", "reg.area"))
Df1$availability <-  sapply(1:nrow(Df1), function(i) Avail[Df1$ID[i],"forest"])
Df1$rel.avail <-  Df1$availability/npatches
M1 <- gam(forest ~ s(tod, bs = "cc") + log(rel.avail+0.001) +
                                      offset(log(rel.avail+0.001)),
          data = Df1, family = "binomial")

Df2 <- subset(Df, habitat %in% c("openland", "reg.area"))
Df2$availability <-  sapply(1:nrow(Df2), function(i) Avail[Df2$ID[i],"openland"])
Df2$rel.avail <-  Df2$availability/npatches
M2 <- gam(openland ~ s(tod, bs = "cc") + log(rel.avail+0.001) +
                                          offset(log(rel.avail+0.001)),
          data = Df2, family = "binomial")
```

The models can be used to give predictions about the odds ratios that forest (or openland) will be selected over regeneration area over time of day and over the range of availability.

```
TOD <- 0:24

rel.avail_reg.area <- mean(Avail[,"reg.area"]/npatches)
Pdf1 <- expand.grid(tod = TOD, rel.avail = seq(0,1-rel.avail_reg.area,length = 50))
Pdf2 <- expand.grid(tod = TOD, rel.avail = seq(1-rel.avail_reg.area, 0,length = 50))
Pdf <- cbind(Pdf1, rel.avail2 = Pdf2$rel.avail)
Pdf$OddsRatios1 <- predict(M1, newdata = Pdf1, type = "link")
Pdf$OddsRatios2 <- predict(M2, newdata = Pdf2, type = "link")
```

The odds ratios are used to obtain real probabilities by applying the multinomial logit link.

```
Pdf$Probability1 <- exp(Pdf$OddsRatios1)/(1+exp(Pdf$OddsRatios1)+exp(Pdf$OddsRatios2))
Pdf$Probability2 <- exp(Pdf$OddsRatios2)/(1+exp(Pdf$OddsRatios1)+exp(Pdf$OddsRatios2))
```

Finally, we can visualize the predicted proportional use of the land cover types.

```
G1 <- ggplot(subset(Pdf, tod %in% 0:12) , aes(x = rel.avail, y = Probability1,
                                              group = as.factor(tod),col = as.factor(tod))) +
        geom_line() + geom_abline(intercept = 0) + ylim(0,1) +
        ggtitle("Estimated proportional use of forest \n based on  discrete choice model") +
        guides(col=guide_legend(title="time of day"))
G2 <- ggplot(subset(Pdf, tod %in% 0:12) , aes(x = rel.avail2, y = Probability2,
                                              group = as.factor(tod), col = as.factor(tod))) +
          geom_line() + geom_abline(intercept = 0) + ylim(0,1) +
```

```
        ggtitle("Estimated proportional use of openland \n based on discrete choice model") +
        guides(col=guide_legend(title="time of day"))

ggarrange(G1,G2 )
```

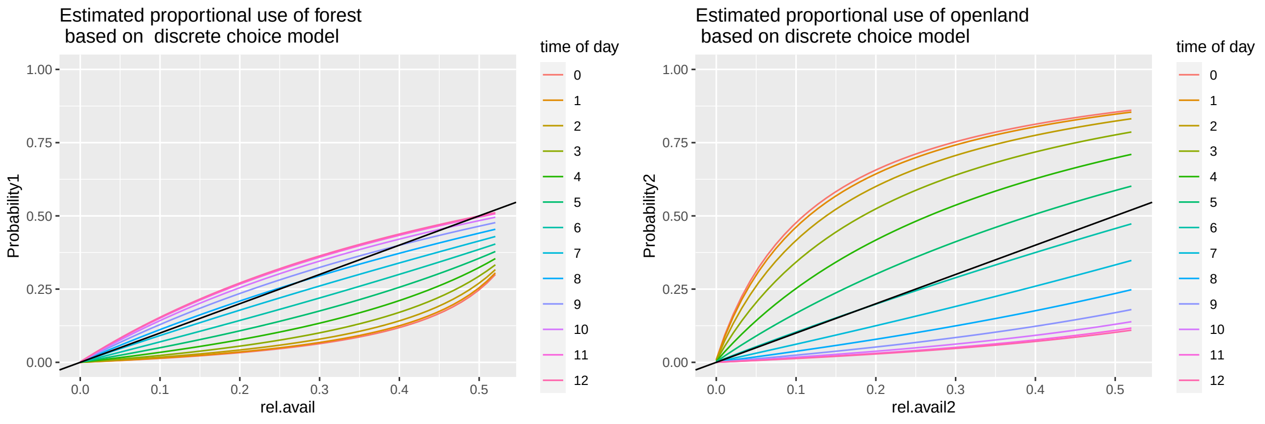


## Fitting Holling types (2nd time)

Now we can determine the optimal Holling type and calculate the associated parameter for our curves and plot them.

```
HT_pred1 <- lapply(TOD ,function(t)
            getholtype(Pdf[Pdf$tod == t,c("rel.avail", "Probability1")],
                                                 type = HT_sim1[t+1,"type"]))
HT_pred2 <- lapply(TOD ,function(t)
            getholtype(Pdf[Pdf$tod == t,c("rel.avail2", "Probability2")],
                                                  type = HT_sim2[t+1,"type"]))

H_func <- list(H1, H2, H3)

htp_pred1 <- lapply(HT_pred1,function(X){
     H_func[[X$type]](rel.avail, c(X$a, X$b))
    })

htp_pred2 <- lapply(HT_pred2,function(X){
     H_func[[X$type]](rel.avail, c(X$a, X$b))
    })

HT_pred1 <- cbind(time_of_day = TOD,do.call(rbind,HT_pred1))
HT_pred2 <- cbind(time_of_day = TOD,do.call(rbind,HT_pred2))
DFH_pred1 <- DFH_pred2 <- expand.grid(rel.avail = rel.avail, tod = TOD)
DFH_pred1$holling <- unlist(htp_pred1)
DFH_pred2$holling <- unlist(htp_pred2)

G1_2 <- ggplot(subset(DFH_pred1, tod %in% 0:12),
              aes(x = rel.avail, y = holling,
                    group = as.factor(tod), col = as.factor(tod))) +
        geom_line() +  geom_abline(intercept = 0) + ylim(0,1) +
        ggtitle("Predicted Holling types for forest") +
        guides(col=guide_legend(title="time of day"))+ ylab("Proportional use")
G2_2 <- ggplot(subset(DFH_pred2, tod %in% 0:12),
```

```
                aes(x = rel.avail, y = holling, group = as.factor(tod),
                    col = as.factor(tod))) + geom_line() +
        geom_abline(intercept = 0) + ylim(0,1) +
        ggtitle("Predicted Holling types for openland") +
        guides(col=guide_legend(title="time of day"))+ ylab("Proportional use")

ggarrange(G1,G2,G1_2,G2_2 )
```

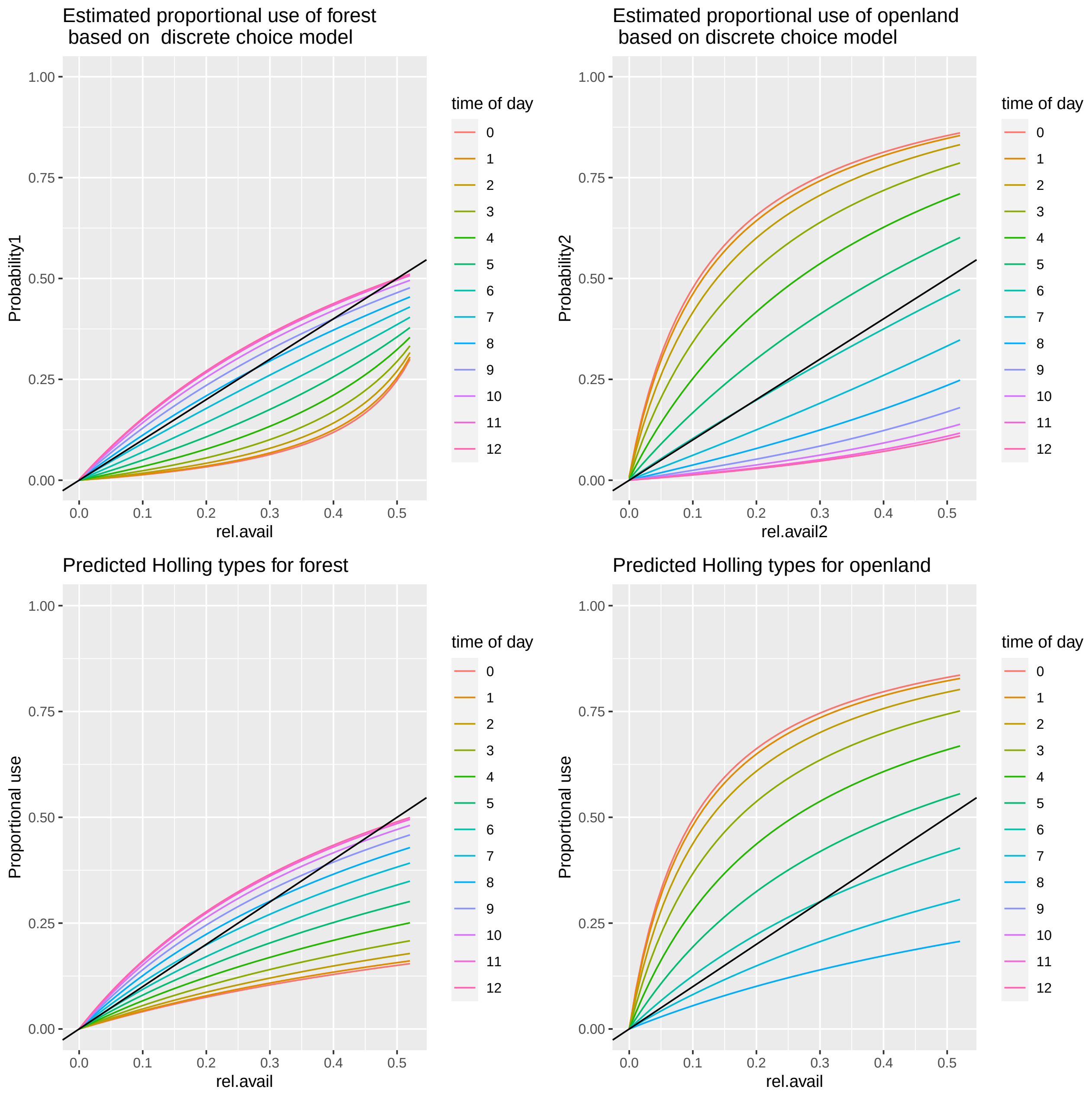


## Checking the model performance

So, let's have a look at the results, namely the estimation of the parameter of our simulation and compare them with the parameter used for the simulation.

```
par_dach <- cbind(time_of_day = TOD, HT_pred1[,c("type","a", "b")],
                  Source = rep("estimated", length(TOD)))
HH <- rbind( cbind(HT_sim1[,c("time_of_day","type","a", "b")] ,
                   Source = rep("simulated", length(TOD))),par_dach)
G1 <- ggplot(data = HH) + geom_point(aes(x=time_of_day, y=a, col = Source, shape = "a"),
                                     size = rep(c(4,2), each = 25)) +
      geom_point(aes(x=time_of_day, y=b, col =Source, shape = "b"),
                        size = rep(c(5,3), each = 25))+
        scale_shape_manual(values=c(17,16)) +
      guides( shape=guide_legend(title="Parameter")) +
      ggtitle("Model check forest") +ylab("Value")

#openland
par_dach <- cbind(time_of_day = TOD, HT_pred2[,c("type","a", "b")],
                  Source = rep("estimated", length(TOD)))
HH <- rbind( cbind(HT_sim2[,c("time_of_day","type","a", "b")] ,
                   Source = rep("simulated", length(TOD))),par_dach)
G2 <- ggplot(data = HH) + geom_point(aes(x=time_of_day, y=a, col = Source, shape = "a"),
                                     size = rep(c(4,2), each = 25)) +
      geom_point(aes(x=time_of_day, y=b, col =Source, shape = "b"),
                  size = rep(c(5,3), each = 25))+
        scale_shape_manual(values=c(17,16)) +
      guides( shape=guide_legend(title="Parameter")) +
      ggtitle("Model check openland")+ylab("Value")

ggarrange(G1,G2)
```

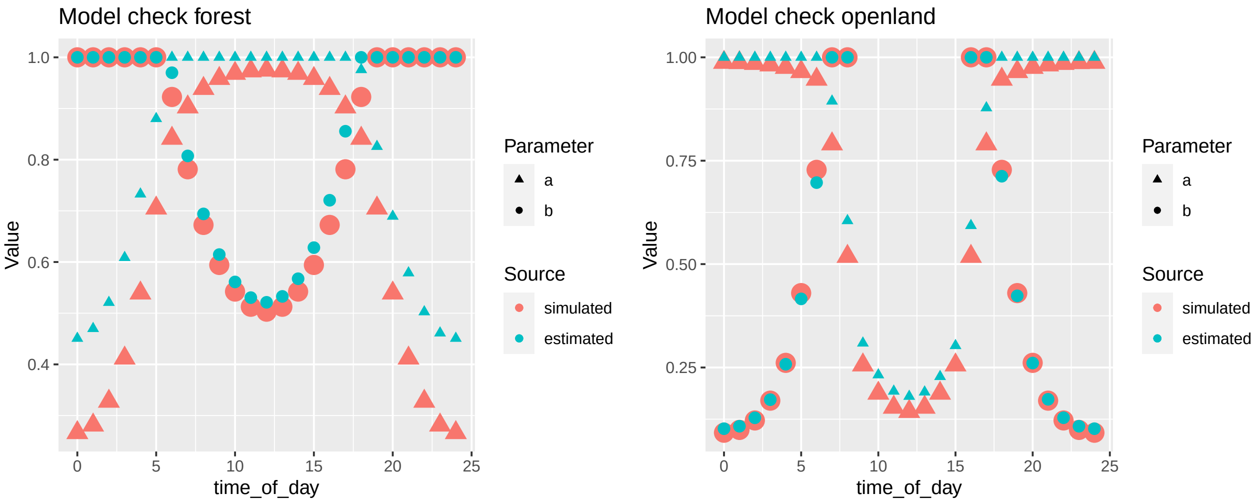


Indeed, we see that in general the parameters are recovered quite well. However, we see that for some areas the deviation is quite strong. We found that the deviation is particularly strong in those areas where the use of both landcover types does not differ much. Consequently, the more different the use of one type is compared to the reference type, the better the estimate.

We encourage the user to play around with the model to get a better feeling for the dependencies.
