# Supplementary material for: Holling meets habitat selection: functional response of large herbivores revisited
Source: Mov Ecol. 2021 Sep 6;9:45. doi: 10.1186/s40462-021-00282-6 (PMC8422736; doi:10.1186/s40462-021-00282-6)
Supplement: Supplementary file 3 — Additional file 3. Vignette to R package FunResp. [file 40462_2021_282_MOESM3_ESM.html]

FunResp: Multicategory logit models for habitat selection including functional response


# FunResp: Multicategory logit models for habitat selection including functional response

#### Claudia Dupke

#### 2020-11-25

Abstract

Discrete choice models are implemented as mixed-effects baseline-category logit models to analyze the variation of habitat category use of moving animal over various temporal scales ( see an application here Dupke et al. 2017). Specifically, changes in habitat use with respect to habitat availability can be investigated leading to functional response curves that further can be fitted to Holling’s types.

- Getting FunResp
- Getting started
- Setting up Multicategory logit models
- Functional response
  - Holling’s type
- Prediction of habitat use over temporal scales

## Getting FunResp

from Github

```
 install.packages("devtools")
 library(devtools)
 install_github("cdupstats/FunResp")
```

## Getting started

Load packages and data

```
   library(FunResp)
   library(mgcv)
   data(Data)
   data(Landscapegrid)
```

The provided toy dataset consists of GPS-telemetry data of three adult roe deer recorded in 2009/2010 in the Bavarian Forest Nationalpark, Germany. Explore the data (for your own data use other packages for an in-depth analysis).

```
   image(Landscapegrid,attr="habitats")
   points(Data$x,Data$y,col=as.numeric(Data$burst)+10)
```

```
   Data$month <- as.numeric(format(Data$date,"%m"))
   Data$hour <- as.numeric(format(Data$date,"%H"))
   table(Data[,c("month","burst")])
```

```
##      burst
## month Eduard_2010 Elfriede_2009 Emily_2010
##     5         119            97         57
##     6         222            90         55
##     7          83           155         57
##     8           0           117         57
```

What you need to apply multicategory models is a representation of the study area as categories, e.g. habitat types.

```
  Data$habitat  <- over(SpatialPoints(cbind(Data$x,Data$y)),Landscapegrid)$habitat
  Data$habitat  <- relevel(Data$habitat, ref="old mixed")
```

## Setting up Multicategory logit models

Get the availability of each habitat type in the monthly home ranges.

```
   par(mfrow=c(3,4),mar=c(1,1,1,1),oma=c(1,1,1,1))
   HRavail <- HRti(Data,varti="month",Landscapegrid,plot=TRUE)

   Data$month_f <- as.character(floor(Data$month))
   Data2 <- cbind(Data,HRavail[apply(Data[,c("burst","month_f")],1,paste,collapse="."),-c(1:2)])
   Data2$avail <- unlist(sapply(1:nrow(Data2),function(i)   HRavail[apply(Data2[i,c("burst","month_f")],1,paste,collapse="."),as.character(unlist(Data2[i,"habitat"]))]))
   Data2$relavail <- Data2$avail/Data2$total
```

Prepare the model, by adding a dummy variable for the random effect and set the model formula. Basically, you can choose any model formula for each habitat type (see examples behind the #).

```
   Data2$dum1 <- 1 #dummy variable
   # Run models (for all habitats but the baseline category)
   # set the model first
    f1 <- formula(present~offset(log(rel.avail))
                           +s(hour,bs="cp") #+s(month,bs="cp")#+te(month,hour,by=sex,bs="cp")
                           + rel.avail # s(rel.avail,bs="ps")#+rel.avail*season
                           +s(burst,by=dum1,bs="re"))
```

Check whether you have enough data for each habitat type. Here, we use a very small data set and I remove all habitats with less than 11 recordings. Then relevel the factor variable of the habitat types to the most common reference type, here: “old mixed”

```
  nn <- table(Data2$habitat)
  habitats <- names(nn)[which(nn>10)] #take only those with more than 10 recordings
  Data2 <- subset(Data2,habitat %in% habitats)
  Data2$habitat  <- factor(relevel(Data2$habitat, ref="old mixed")) #make sure that baseline habitat is at first position like relevel(Data$habitat, ref="old mixed")
```

Estimate the multicategory logit models, one for each habitat except the baseline category and plot the results. This is just a small toy dataset for the purpose of demonstration. Therefore, the patterns here are somewhat disappointing.

```
   MM <- multicat1(Data2,HRavail,f1,sp=c(1,2,1))
   names(MM) <- habitats[-1]
   par(mfrow=c(3,6),mar=c(1,1,1,1),oma=c(1,1,1,1))
   invisible(lapply(MM,plot))
```

## Functional response

Now you can use the model to make predictions for functional repsonse for each habitat type. First, set the data for predictions as a sequence of values for relative availability for each habitat (make sure that it does not exceed the range of the observed data as we should avoid extrapolation).

```
seqRA <- sapply(habitats[-1],function(ty) try(seq(min(exp(MM[[ty]]$model$"offset(log(rel.avail))")),min(quantile(exp(MM[[ty]]$model$"offset(log(rel.avail))"),.95),0.9), length=100)))
seqRA <- cbind(seqRA,"old mixed"=seq(0.001,0.5,length=nrow(seqRA)))
```

You also need to fix the values for availability for all available habitats as these are needed for predicting the use of a focal habitat. As you remember, we assume that the animal can choose between different habitats at the same time (discrete choice) and therefore the probability of use of other habitats affect the probability of use of the focal habitat. As we told our model, the probability of use depends on the availability of a habitat. I recommend using the mean and transform the values so that availabilities of all habitats sum to 1.

```
#mean availability for each habitat
UP <- unique(Data2[,c("burst","habitat","relavail")])
ma3 <- aggregate(UP$relavail,by=list(UP$habitat),mean)
meanavail <- ma3$x
names(meanavail)  <- ma3$Group.1
meanavail <- meanavail/sum(meanavail)
```

As your model varies over time you have to fix the values for time and all other covariates included in the model to make the prediction.

```
hour <- 0
#start predictions
Preds <- lapply(habitats[-1],makpred_P,hoset=hour,seqRA=seqRA,meanavail=meanavail,Mod=MM)
names(Preds) <- habitats[-1]
#plot predictions
par(mfrow=c(2,3),mar=c(4,4,1,1),oma=c(2,2,2,2))
invisible(lapply(1:length(Preds),function(X) plot(Preds[[X]][,"relavail"],Preds[[X]][,"fit"],main=names(Preds)[X],type="l",ylab="Proportion of use",xlab="Relative availability")))
```

### Holling’s type

The estimated functional response curve are now linked to Holling’s types: For type I: \(h\_I(x)=a x\), where \(x\) is the relative availability of a habitat, a value between 0 and 1, and \(a\) the proportionality factor; for type II: \(h\_{II} (x)=\frac{ax}{b+x}\) and for type III: \(h\_{III} (x)=\frac{ax^2}{b^2+x^2}\). The optimisation routine minimises the residual sum of squares between the values of \(h\_I(x)\), \(h\_{II}(x)\) and \(h\_{III}(x)\) and the values of the curve estimated from the multicategory logit models by finding optimal values for \(a\) and \(b\). The R function of the R-package was used for this purpose, with the values of \(a\) and \(b\) limited to 0 and 1 for \(h\_{II}\) and \(h\_{III}\). The Holling type with the smallest residual sum of squares is considered to best explain the shape of the functional response curve.

```
HT <- lapply(1:length(Preds), function(X) optimHolling(Preds[[X]][,"relavail"],Preds[[X]][,"fit"]))
names(HT) <- names(Preds)
HTty <- do.call(rbind, HT)

par(mfrow=c(2,3),mar=c(4,4,1,1),oma=c(2,2,2,2))
invisible(lapply(1:length(Preds),function(ty) {
           x <- Preds[[ty]][,"relavail"]   
           plot(x,Preds[[ty]][,"fit"],main=names(Preds)[ty],type="l",ylab="Proportion of use",xlab="Relative availability", xlim=c(0, max(x)), ylim = c(0,max(Preds[[ty]][,"fit"])))
           if(HTty[ty,"type"]==1){
               lines(x,Holling1(x,HTty[ty,"a"]),col="red", lty=3, lwd=3)
           }
           if(HTty[ty,"type"]==2){
               lines(x,Holling2(x,HTty[ty,"a"],HTty[ty,"b"]),col="red",lty=3,lwd=3)
           }
           if(HTty[ty,"type"]==3){
               lines(x,Holling3(x,HTty[ty,"a"],HTty[ty,"b"]),col="red",lty=3,lwd=3)
           }
           abline(a=0,b=1,col="darkgrey")
           mtext(paste("Hollingtype",c("I","II","III","IV")[HTty[ty,"type"]],"\n a=",round(HTty[ty,"a"],2),"\n b=",round(HTty[ty,"b"],2)),side=1,line=-2,adj=0.9, col = "red")
       }))
```

One more example, based on real data:

```
data(FemalesNight)
par(mfrow=c(4,3),mar=c(4,4,1,1),oma=c(2,2,2,2))
invisible(lapply(1:length(FemalesNight),function(X) plot(FemalesNight[[X]][,"RA"],FemalesNight[[X]][,"fit"],main=names(FemalesNight)[X],type="l",ylab="Proportion of use",xlab="Relative availability")) )

HT <- lapply(1:length(FemalesNight), function(X) optimHolling(FemalesNight[[X]][,"RA"], FemalesNight[[X]][,"fit"]))
names(HT) <- names(FemalesNight)
HTty <- do.call(rbind, HT)

par(mfrow=c(4,3),mar=c(4,4,1,1),oma=c(2,2,2,2))
```

```
invisible(lapply(1:length(FemalesNight),function(ty) {
           x <- FemalesNight[[ty]][,"RA"]  
           plot(x,FemalesNight[[ty]][,"fit"],main=names(FemalesNight)[ty],type="l",ylab="Proportion of use",xlab="Relative availability", ylim = c(0,max(FemalesNight[[ty]][,"fit"])))
           if(HTty[ty,"type"]==1){
               lines(x,Holling1(x,HTty[ty,"a"]),col="red", lty=3, lwd=3)
           }
           if(HTty[ty,"type"]==2){
               lines(x,Holling2(x,HTty[ty,"a"],HTty[ty,"b"]),col="red",lty=3,lwd=3)
           }
           if(HTty[ty,"type"]==3){
               lines(x,Holling3(x,HTty[ty,"a"],HTty[ty,"b"]),col="red",lty=3,lwd=3)
           }
           abline(a=0,b=1,col="darkgrey")
           mtext(paste("Hollingtype",c("I","II","III","IV")[HTty[ty,"type"]],"\n a=",round(HTty[ty,"a"],2),"\n b=",round(HTty[ty,"b"],2)),side=1,line=-2,adj=0.9, col = "red")
       }))
```

## Prediction of habitat use over temporal scales

Now, you can use the model to predict habitat use over the range of covariates used in your model, e.g. time of day (hour). Keep in mind, that this is just a small toy data set for the purpose of demonstration. Therefore, the patterns here are somewhat disappointing. The use of the reference (or baseline) type has to be calculated seperately.

```
hour <- 0:23
RA <- matrix(rep(meanavail,each=length(hour)),nrow=length(hour),dimnames=list(c(),c(names(meanavail))))
Preds <- lapply(habitats[-1],makpred_P,hoset=hour,seqRA=RA,meanavail=meanavail,Mod=MM)
names(Preds) <- habitats[-1]
par(mfrow=c(2,4))
invisible(lapply(1:length(Preds),function(X) plot(Preds[[X]][,"hour"],Preds[[X]][,"fit"],type="l",ylim=c(0,.4),main=names(Preds)[X],ylab="Proportion of use",xlab="Time of day (Hour)")))

# prediction for baseline category
predhour <- do.call(rbind,lapply(Preds,function(X) X[,"fit"]))
pred_base <- 1-apply(predhour,2,sum)

plot(hour,pred_base,type="l",ylim=c(0,.4),main=habitats[1],ylab="Proportion of use",xlab="Time of day (Hour)")
```
